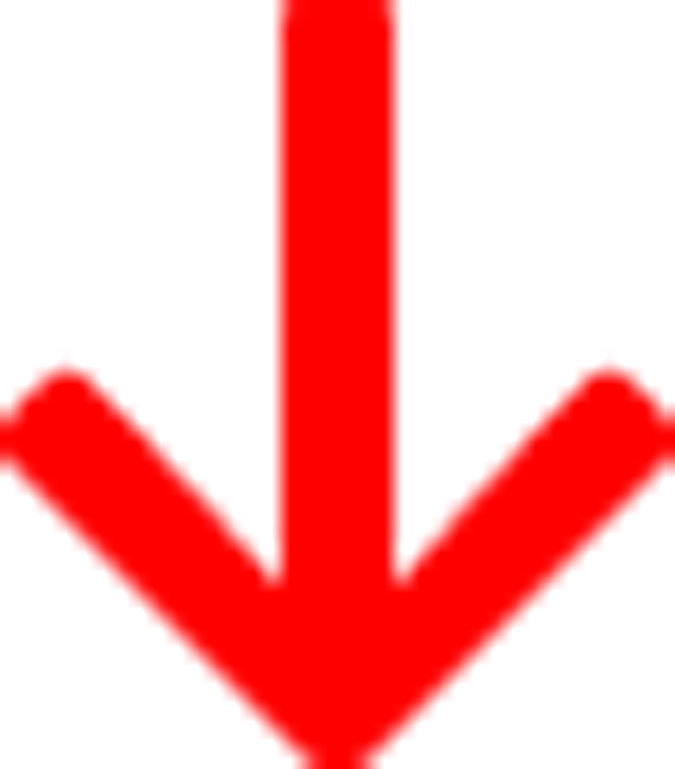# Insights into the Transcriptional Architecture of Behavioral Plasticity in the Honey Bee *Apis mellifera*

**DOI:** 10.1038/srep11136

**Published:** 2015-06-15

**Authors:** Abdullah M. Khamis, Adam R. Hamilton, Yulia A. Medvedeva, Tanvir Alam, Intikhab Alam, Magbubah Essack, Boris Umylny, Boris R. Jankovic, Nicholas L. Naeger, Makoto Suzuki, Matthias Harbers, Gene E. Robinson, Vladimir B. Bajic

**Affiliations:** 1 Computational Bioscience Research Center, Computer, Electrical and Mathematical Sciences and Engineering Division, King Abdullah University of Science and Technology (KAUST), Thuwal 23955-6900, Saudi Arabia; 2 Departments of Entomology and Institute for Genomic Biology, Urbana, IL 61801; and Neuroscience Program, University of Illinois at Urbana–Champaign, Urbana, IL 61801; 3 Lumenogix Inc., 2935 Rodeo Park Drive East, Santa Fe NM, 87505, USA; 4 DNAFORM Inc., Leading Venture Plaza-2, 75-1, Ono-cho, Tsurumi-ku, Yokohama City, Kanagawa, 230-0046, Japan; 5 RIKEN Center for Life Science Technologies, Suehiro-cho, Tsurumi-ku, Yokohama City, Kanagawa, 230-0045, Japan

## Abstract

Honey bee colonies exhibit an age-related division of labor, with worker bees performing discrete sets of behaviors throughout their lifespan. These behavioral states are associated with distinct brain transcriptomic states, yet little is known about the regulatory mechanisms governing them. We used CAGEscan (a variant of the Cap Analysis of Gene Expression technique) for the first time to characterize the promoter regions of differentially expressed brain genes during two behavioral states (brood care (aka “nursing”) and foraging) and identified transcription factors (TFs) that may govern their expression. More than half of the differentially expressed TFs were associated with motifs enriched in the promoter regions of differentially expressed genes (DEGs), suggesting they are regulators of behavioral state. Strikingly, five TFs (nf-kb, *egr*, *pax6*, *hairy*, and *clockwork orange*) were predicted to co-regulate nearly half of the genes that were upregulated in foragers. Finally, differences in alternative TSS usage between nurses and foragers were detected upstream of 646 genes, whose functional analysis revealed enrichment for Gene Ontology terms associated with neural function and plasticity. This demonstrates for the first time that alternative TSSs are associated with stable differences in behavior, suggesting they may play a role in organizing behavioral state.

Due to its extensive behavioral repertoire and highly social lifestyle, the European honey bee (*Apis mellifera*) has been utilized as an ethological model for decades. More recently, the publication of the honey bee genome^1^, quantitative trait locus analyses^2^, and transcriptomic studies^3^ have positioned the honey bee at the forefront of efforts to understand the relationship between genes, the environment, and complex behavior. Adult worker honey bees exhibit behavioral maturation and transition between discrete sets of tasks as they age^4^. Bees perform tasks in the hive for the first 2–3 weeks of their 6–7 week adult life, such as cleaning or building new honeycomb and tending to (“nursing”) the brood. They then transition to working outside the hive, guarding its entrance or foraging for food and other resources. While this behavioral maturation has a strong age-related foundation, bees are also able to respond to changing colony conditions by accelerating, delaying or even reversing their trajectory. This behavioral plasticity is influenced by a complex of factors including genotypic background, colony demography, nutrition, and the availability of colony resources^4^. It is also mediated by specific endocrine factors and neuromodulators, and associated with changes in the expression of thousands of genes in the brain, some of which have causal effects on behavior^3^. As a result, transcriptomic analyses of behavioral maturation in honey bees have led to fundamental insights about how genotype and the environment act on the brain transcriptome to regulate behavior^3^.

Two particular behavioral states in honey bees, nursing and foraging, are often used to characterize the relationship between behavioral maturation and the transcriptome due to the well-characterized and distinct suites of behaviors that each entails. While the social, neuroendocrine, physiological, molecular, and genetic influences mediating these states have been elucidated in numerous studies^3^, the transcriptional regulatory architecture in the brain underlying and connecting these maturational determinants remains largely unknown. A brain transcriptional regulatory network (TRN) derived from co-expression data collected in a large set of microarray studies revealed that a small number of TFs were predicted to reliably regulate the vast majority of differentially expressed genes (DEGs) in the brain^5^. Similarly, examining the *cis*-regulatory logic underlying motifs present in the promoters and enhancers of DEGs revealed that specific combinations of motifs (many of which are binding sites for TFs identified in the above-mentioned brain TRN^5^) were reliably associated with the differential expression of maturation-related genes in the brain^6^. Together, these results strongly suggest that a set of key TFs are responsive to maturational determinants and regulate definable gene modules to govern patterns of behavior. A comprehensive understanding of the manner in which these TFs contribute to behavioral state is thus essential to furthering our understanding of how behavior is organized.

As can be seen, there is great interest in elucidating the genome-scale TRNs underlying behavior^5^,^6^,^7^,^8^,^9^. However, because bioinformatics and experimental methods for identifying potential *cis*-regulatory sites upstream of the transcriptional start site (TSS) can be unreliable or difficult, respectively^10^,^11^, an ideal approach is to use a combination of methods to increase the robustness of inferences made about a network’s regulatory architecture. Since recent studies have highlighted the fact that a surprising proportion of potential binding sites in the promoter’s immediate vicinity exert functional influences on gene expression^12^, the region surrounding the TSS may provide particularly valuable insights about the identity of the TFs regulating a gene. Indeed, it appears that TF binding at the promoter is so vital that regulator-target interactions during development can be conserved over vast evolutionary distances^13^.

Transcriptomic techniques based on cap analysis of gene expression (CAGE) allow for high-throughput deep sequencing of the 5’-ends of mRNA transcripts to identify a gene’s TSS as well as promoter features downstream of the start site by selectively enriching and sequencing the region immediately downstream of the 5’ methylguanosine cap^14^. This allows one to spatially restrict motif finding to *cis*-regulatory modules that are actively co-transcribed with the target gene, and thus likely to be biologically relevant^15^. These modules can then be used to create a high resolution map of the transcriptional start sites upstream of actively transcribed genes^16^.

In order to determine how TFs (as well as promoter and TSS characteristics) might contribute to behavior, we used CAGEscan^17^ to examine the transcriptional regulatory architecture in the brain underlying behavioral maturation. The large quantities of RNA required to perform traditional CAGE and SAGE techniques preclude the analysis of individual bee brains, a critical factor in accurately characterizing nuanced transcriptomic changes associated with behavioral state. CAGEscan, however, is a variant of the nanoCAGE technique and is designed expressly for promoter characterization from small quantities of input RNA^17^. Mapping CAGEscan reads to a reference genome allows for accurate identification of TSS and the related promoter and 3’ region of the expressed gene. CAGEscan thus permits one to detect subtle changes in gene expression and link them to promoter characteristics such as motif composition of the promoter and TSS. With CAGEscan it is also possible to utilize paired-end-reads to provide additional information on the 3’end of the DNA fragments within the library. The additional 3’-end reads are used to improve mapping to the reference genome and to more accurately associate 5’-end reads to genes, and allowing for the discovery of novel promoter regions and TSSs. Here we report on the first comprehensive use of CAGEscan and mapping of TSS followed by promoter analysis of honey bee behavioral maturation.

Alternative TSSs are a pervasive feature in eukaryotic genomes, and a growing body of evidence indicates that they may play a vital role in gene regulation^18^. While they can arise from distinct promoter regions clearly separated by long stretches of sequence, alternative TSSs can also occur close to each other within the same promoter region; even subtle alterations in a gene’s TSS have been associated with changes in the expression of downstream genes in *Drosophila melanogaster*^19^,^20^ and mammals^21^. CAGE-based techniques have already made valuable contributions to our understanding of transcription in model organisms such as, the fruit fly^19^,^20^, zebrafish^22^, and human and mouse^15^,^23^,^24^, and specifically in the nervous system, where alternative TSSs appear to play a role in establishing developmental^25^ and region-specific^26^ gene expression patterns. However, the potential relevance of alternative TSSs in organizing behavior has, to our knowledge, not been addressed in any organism. A previous characterization of promoter usage at the transcriptome level using 5’ LongSAGE and expressed sequence tags found that there was evidence for TSS variability in nearly half of the genes transcribed in the head of male bees^27^, suggesting that promoter and TSS usage may also play a vital role in the regulatory systems underlying behavioral maturation.

Using CAGEscan to associate differentially expressed TFs with motif enrichment in the promoter region of DEGs, we were able to infer the identity of putative regulators of DEGs in specific behavioral contexts. Moreover, the identification of many of these TFs in previous analyses^5^ suggests that they may play a role as regulators of not only individual genes, but of the behavioral state itself. If so, they would represent crucial links between the transcriptomic architecture and behavior. Finally, we used CAGEscan to accurately detect TSSs for every expressed gene, which enabled us to discover differences in TSS usage in different behavioral contexts. For the first time, this implicates alternative TSS usage as a potential mechanism regulating the transcriptomic changes underlying behavioral maturation.

## Results and Discussion

### Read Mapping and Gene Expression

**Figure 1:**
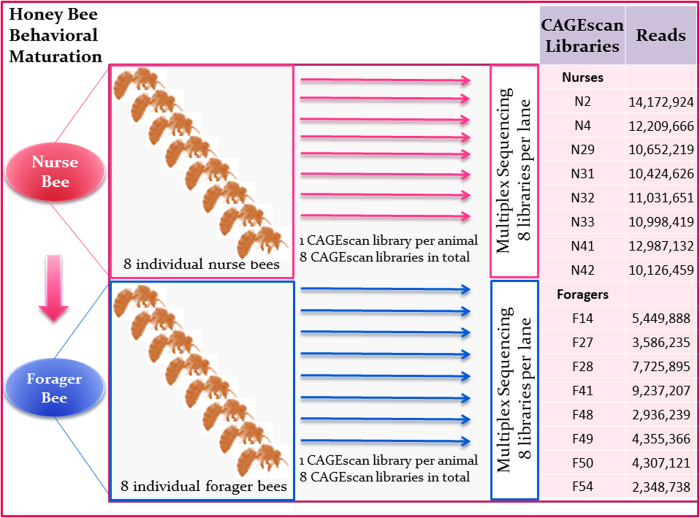
Overview of library preparation and sequencing. CAGEscan libraries were generated from total RNA extracted from individual brains of 8 nurses and 8 foragers. Barcoded cDNAs were pooled into two lanes and sequenced on an Illumina HiSeq2000. The resulting reads can be viewed in the table on the right.

To elucidate the regulatory networks and TFs underlying behavioral plasticity in honey bees, we prepared CAGEscan libraries from the brains of individual nurses and foragers. Libraries were pooled into two groups of eight (corresponding to nurses and foragers) for sequencing on an Illumina platform (Fig. 1), and sample-specific barcodes were used to differentiate between individuals. Initial sequencing of the forager samples revealed a low number of reads relative to standard RNAseq protocols. Since this deficiency in reads was likely due to the sequencing protocol rather than the quality of the RNA (Supplementary Table S1), the input cDNA of the nurse samples was increased to compensate. A total of 102,568,069 and 67,921,806 paired-reads were obtained from the sequencing of the nurse and forager samples, respectively; after filtering for read quality, 92,603,096 and 39,946,689 paired-reads were retained (Fig. 1, Supplementary Table S2). 63% and 59% of nurse and forager reads, respectively, could be mapped to v4.5 of the honey bee reference genome (Supplementary Table S3), and were then processed for mapping quality (Supplementary Table S4). 83% to 90% of the CAGE tags from each sample could be mapped to genes in the honey bee genome (Supplementary Table S5), and we were able to associate CAGE tags with 13,111 genes. After normalizing and filtering the genes (see Methods), 12,453 of the 15,314 genes in OGSv3.2 (81.3%) had measurable levels of expression (Table 1). Despite the low quantity of read counts in our samples relative to traditional RNAseq studies, plotting saturation curves indicated that the degree of coverage was adequate to capture genes with a low level of expression, even in the forager samples (Supplementary Fig. S1). For additional measures of read quality and distribution, see Supplementary Tables S1–S5.

**Figure 2:**
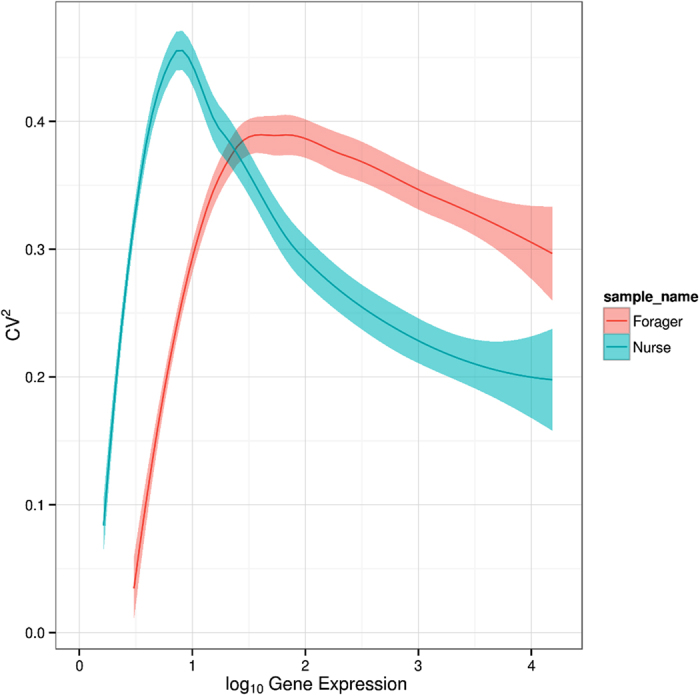
The squared coefficient of variation (CV2) in per-gene expression for foragers and nurses. The x-axis is the log_10_ normalized per-gene expression level and the y-axis is the squared coefficient of variance (CV^2^). It is apparent that variability in gene expression within foragers is higher than nurses for most genes, yet not for genes with a low level of expression (which should be the most prone to variation arising from technical artifacts).

Comparing the per sample biological coefficient of variation (Supplementary Fig. S2) and per gene squared coefficient of variation (Fig. 2) revealed that there was a substantially higher degree of within-group variation in gene expression among foragers than nurses (p-value < 1.0e-300, Wilcoxon Rank Sum Test). Although this increase in variance could theoretically be due to the lack of read coverage in forager samples relative to nurses (Supplementary Table S2), we minimized the impact of coverage-related biases by normalizing gene expression (see Methods). Moreover, if the variance was a result of low coverage, one would expect genes with a low level of expression to be the most adversely affected, and thus have the highest variance. However, this does not appear to be the case (Fig. 2), indicating that read count did not contribute significantly to variation in gene expression or, by extension, differential gene analyses. It is possible, then, that the discrepancy in variation is a biologically relevant phenomena and may reflect the fact that the foragers have to respond to a far more diverse set of stimuli (samples were collected on their return trip) and adapt to more variable conditions (i.e., outside environment and varying floral conditions) than do the hive-bound nurses. Although no prior study has explicitly compared nurse and forager variability in gene expression, forager variability has itself been the focus of other studies, which found that differences in experience, motivational state and environmental exposure can lead to distinct neurotranscriptomic states^3^,^28^.

**Figure 3:**
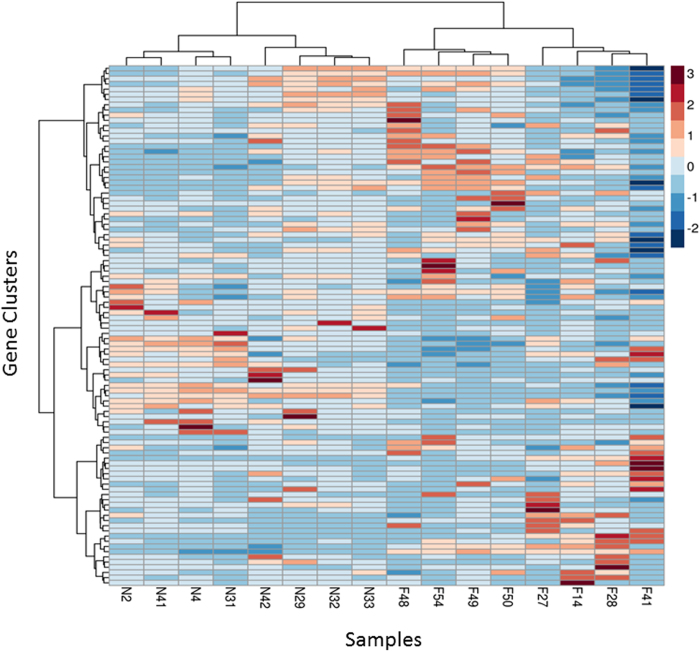
Hierarchical clustering of the brain gene expression profiles of nurse and forager honey bees. Clustering was performed using Ward’s method. Rows correspond to 100 clusters obtained from 12,453 genes by the k-means algorithm and columns represent nurse (‘N’) and forager (‘F’) samples. The scale bar indicates the z-scores of gene expression values, such that highly expressed genes are depicted in 
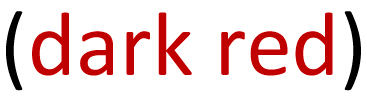
 while genes with low levels of expression are depicted in 
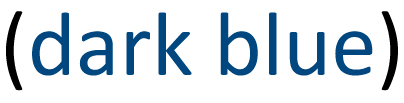
. A heatmap showing the hierarchical clustering of all 12,453 genes without K-Means clustering is provided in (Supplementary Fig. S2).

Despite the disparity in within-group variance between nurses and foragers, unsupervised hierarchical clustering (Fig. 3A,B, Supplementary Figs S3 and S4) was able to generate two distinct groups of gene expression profiles that correspond directly to the behavioral state of the sampled bee. Hierarchical clustering also revealed discrete within-group clustering of the samples, which may reflect differences in within-group genetic relatedness (despite an average degree of relatedness of 75%), age, or time spent performing a particular activity^28^. A single outlier (sample F41) was identified during this analysis. However, subsequently removing the outlier had little impact on downstream analyses (Supplementary Table S6), and the sample was retained. Overall, these results indicate that CAGEscan was able to recapitulate the strong relationship between neurotranscriptomic and behavioral state observed in previous honey bee microarray studies^29^,^30^,^31^.

### Differentially Expressed Genes

**Figure 4:**
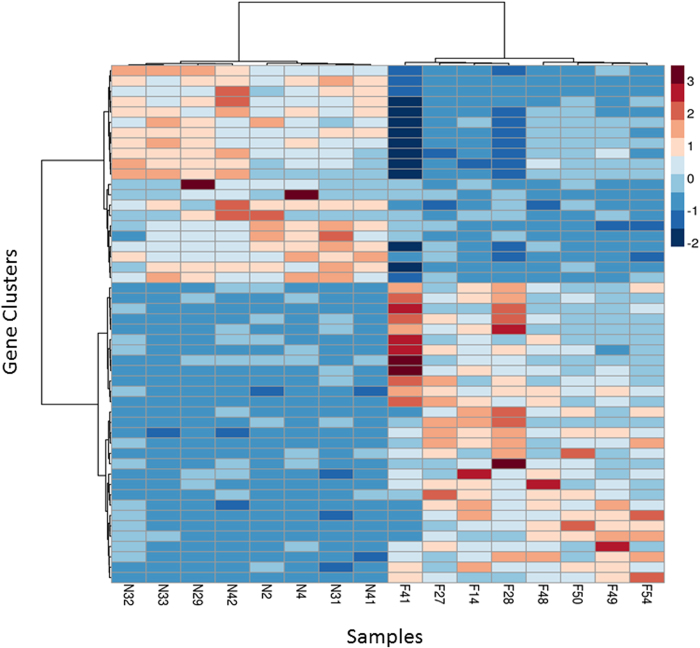
Heatmap for the hierarchical clustering of the differentially expressed brain gene profiles of nurse and forager honey bees. Rows correspond to 50 clusters obtained from 1,058 DEGs by the k-means algorithm. Columns represent samples. The scale bar indicates z-scores of gene expression values, with highly expressed genes depicted in dark red low-expressed genes depicted in dark blue. The heatmap that shows the hierarchical clustering of all 1,058 DEGs without clustering is provided in (Supplementary Fig. S3).

There were 1,058 differentially expressed genes (DEGs) between nurses and foragers (FDR < 0.05, Supplementary Dataset 1, Supplementary Fig. S5). Although the number of DEGs upregulated in both groups is almost identical (534/524 genes in foragers and nurses, respectively), K-Means clustering revealed 29 clusters of upregulated genes in foragers and 21 clusters in nurses (Fig. 4). This suggests that foragers may have greater variation in regulatory patterns, which is consistent with our previous observations on the distribution of variance within the two behavioral groups.

The honey bee brain is surrounded by the hypopharyngeal glands (HPG), making it difficult to dissect the brain without the risk of contamination. Further, because the development of the HPG is intrinsically linked to the maturational state of the bee, contamination can result in systematic biases in gene expression when behavioral maturation is being assessed. Therefore, in order to determine the extent of potential contamination we used RNAseq to obtain an expression profile of nurse and forager HPGs relative to brain tissue. We then compared genes that were upregulated in the HPG to our dataset (Supplementary Table S7). Only 36 of the 1125 genes that were strongly (log_2_ fold-change > 3) upregulated in the HPG were identified as differentially expressed between nurses and foragers, implying that HPG contamination most likely had a minimal impact on the identification of DEGs. Since the potential influence of this contamination appeared to be negligible, no DEGs were removed from subsequent analyses.

### Comparisons with Previous Studies

**Figure 5:**
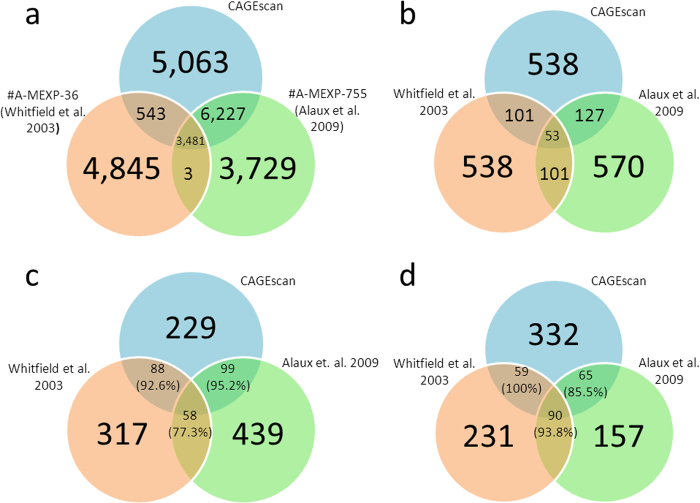
Overlap of DEGs between CAGEscan and previous studies of nurse and forager brain transcriptomes. (a) Represents the relationship between gene models of the newest honey bee genome annotation (OGS 3.2) and the probes that were present on microarray platforms used in previous analyses of honey bee nursing and foraging behavior. Only probes that could be mapped to OGS 3.2 and genes that were present on at least one array (shown in the regions of overlap) were used to assess commonalities between CAGEscan and the two cited studies. (b) Shows the overlap of differentially expressed genes detected by CAGEscan and the two previous microarray based studies of nurse and forager transcription. (c & d) These Venn diagrams display the degree of directional concordance for nurse (c) and forager (d) upregulated genes in the three studies. The areas of overlap represent the number of concordant genes, while the numbers in parentheses indicate the percentage of concordance relative to the number of differentially expressed genes associated with nursing and foraging in each study.

To explore the concordance of these results with previous studies, we compared our data to prior microarray assessments of nurse and forager brain transcriptomes. For consistency, we remapped the microarray datasets to the current official honey bee gene set, OGSv3.2 using BLAT and Bowtie (Fig. 5a). The present CAGEscan and previously published microarray datasets^29^,^31^ show strong similarities in the number of DEGs detected in the brain, with circa 800-900 DEGs for each study (Fig. 5b, Supplementary Dataset 2). Moreover, the DEGs identified in the CAGEscan dataset exhibits a significant degree of overlap with prior microarray assessments of nurse and forager transcriptomes, sharing approximately 150 genes with each previous study (Fig. 5b). Hypergeometric tests indicated that the degree of overlap between the three datasets was modest, but significant (p < 1e-08 for all pairwise comparisons, Bonferroni adjusted). The directional concordance of gene expression changes in the overlapping DEGs was highly consistent, however, with a minimum of 84% concordance (Fig. 5c,d). Moreover, we calculated the Spearman Rank Correlation (r) of the log_2_ fold change of our data and the aforementioned studies, and found robust and reliable correlations in gene expression values between the three studies (r = 0.39, p < 1e-100, comparison of^29^,^31^; r = 0.39, p < 1e-120, comparison of our results and^31^; r = 0.25, p < 1e-125, comparison of our results and^29^).

These results are noteworthy given the differences in sample genetic background, collection protocol, analytical platforms and gene models used in these studies. In particular, models of alternative splicing are not as complete in the honey bee as they are in genetic model organisms, and have shifted considerably with the advent of newer annotations^32^. This could cause isoform specific probes to be misconstrued as indicating a change in overall gene expression when none actually exist. Finally, it should be noted that the degree of concordance between our study and the two array studies was not substantially different from the level of similarity between the two microarray studies themselves, suggesting that discrepancies between these studies may be the result of genetic background or biological noise rather than platform-related differences.

### Gene Ontology Analyses of Differentially Expressed Genes

A Gene Ontology (GO) analysis was performed to explore the functional implications of nurse and forager upregulated genes. Genes upregulated in nurses were found to be enriched for GO terms associated with nucleic acid, lipid and protein metabolism (Supplementary Dataset 3), a result consistent with previous transcriptomic analyses of behavioral maturation^31^. For instance, energy metabolism^33^, oxidoreductase activity^30^, oxidation reduction^6^, glycolysis^6^, and various mitochondrial and ribosomal^34^ components are all GO categories that were identified in both our study and previous studies on maturational determinants (Supplementary Dataset 4). These annotations are particularly relevant, since it is now well established that nutritional physiology has a causal influence on the behavioral state of the honey bee^2^,^3^. Manipulating factors that influence metabolic state such as diet^33^, insulin signaling^35^ and the yolk-protein Vitellogenin affect not only brain gene expression but the rate of behavioral maturation^3^. Indeed, there is evidence of coordinated TRNs in honey bee brain and fat tissues during behavioral maturation, suggesting that brain function and body-wide metabolic changes are intrinsically linked at the level of the transcriptome^36^.

Genes upregulated in foragers were also enriched for some metabolic processes, but there was also far greater diversity in the types of GO terms that characterize forager up-regulated genes, including numerous terms associated with organ development and growth (Supplementary Dataset 3). A closer inspection of these categories reveals that they are composed of genes known to play roles in nervous system development, neuronal function and neural plasticity in *Drosophila melanogaster* (Supplementary Dataset 4). As with nurses, the GO categories linked to foraging are also consistent with previous transcriptomic and informatics based analyses, especially for nervous system development^6^,^37^, synaptic/neurotransmission^5^, receptor signaling pathways^30^, protein kinase activity^28^,^30^, G-protein coupled receptor signaling^28^,^38^, insulin receptor signaling^34^, protein folding^6^,^28^,^30^, and response to heat^6^,^28^. These results may reflect the highly demanding cognitive tasks that foraging honey bees must perform relative to nurses related to navigation, manipulating flowers, and forming spatiotemporal memories of different foraging sites^34^, though experiments that directly manipulate the effects of these factors on the performance of foraging activities are still limited.

### Transcription Factors Identified as Key Regulators of Behavioral Maturation

In total, 250 orthologous TFs were identified by sequence similarity. 26 of these TFs were differentially expressed, with 4 upregulated in nurses and 22 upregulated in foragers (Supplementary Dataset 5, Supplementary Fig. S6 and S7). Additionally, more than half of the differentially expressed TFs had DNA binding motifs that were statistically enriched in the promoter regions of differentially expressed genes (Table 2), strongly suggesting they are part of the regulatory architecture underlying behavioral state.

**Figure 6:**
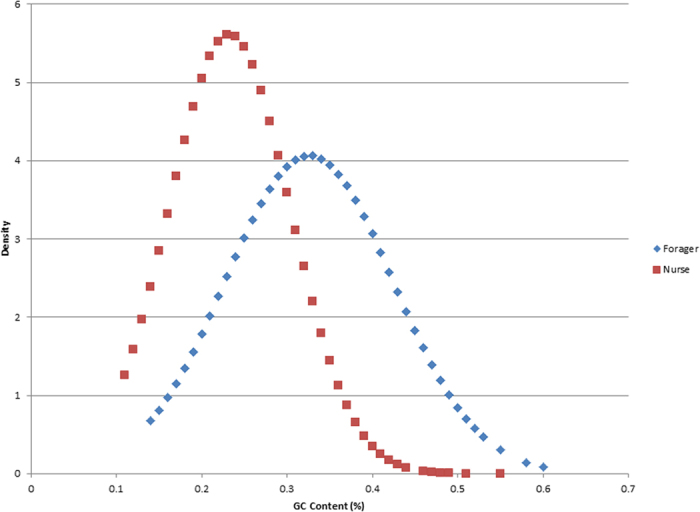
G/C content distribution for the promoters of DEGs. Promoters associated with Forager upregulated genes have a significantly higher percentage of G/C nucleotides than those associated with Nurse upregulated genes, which could bias motif enrichment analyses.

Previous studies have indicated that the G/C content of promoter regions can have a dramatic impact on motif identification^37^. To ascertain whether our analysis was influenced by this bias, we compared the relative G/C content of promoters associated with forager and nurse upregulated genes. We found that the promoters of forager upregulated genes were indeed significantly enriched for G/C nucleotides compared to those of nurse upregulated genes (p-value < 1.0e-50, Wilcoxon Rank Sum Test, Supplementary Dataset 6, Fig. 6). Since our initial analysis used nurse and forager promoters as background sets when assessing enrichment, a difference in C/G content between these groups could adversely affect these findings. In order to verify that our motif enrichment data were not compromised, we performed two additional analyses using alternative backgrounds consisting of 1) all predicted promoters in OGS v3.2 or 2) randomized portions of the bee genome. Since the motifs of only two TFs were altered in these new analyses (Table 3), we conclude that C/G bias we detected exerted a minimal influence on our analysis.

To determine whether each of the 15 putative regulators of behavioral state might serve as activators or repressors of their target genes, we compared the expression patterns of the TFs themselves with the patterns of the genes they were predicted to regulate. Eight putative regulators had motifs that were enriched in the promoters of genes upregulated in the same behavioral context (Table 3) suggesting that they have an activating influence on their targets. Conversely, five putative regulators have a reciprocal relationship with their predicted targets, suggesting that they are serving as repressors of these genes. Finally, the last two TFs had motifs that were enriched in the promoters of both forager and nurse upregulated genes relative to all annotated promoters in the genome, suggesting they may have bivalent regulatory functions. Remarkably, these predictions are largely consistent with the known functions of orthologous genes in other organisms and contexts (Table 3). That being said, it should be noted that these functions may not correspond with canonical descriptions of the TF in question, as some of these TFs have been documented to possess dual activator and repressors functions in different contexts.

Two of these putative regulators, Creb1 and NF-κB, have previously been identified as potential regulators of behavioral maturation in both a reconstruction of the honey bee brain TRN^5^ and in motif distribution analyses of the regulatory regions of genes associated with behavioral maturation^6^. Both Creb1 and NF-κB have also been experimentally shown to play vital roles in regulating neural plasticity^39^,^40^,^41^,^42^, in addition to their involvement in other biological processes. Intriguingly, the genes of several TFs that interact with Creb1 were found to be differentially expressed, including *atf3* and *usf1.* Like Creb1, Atf3 is a critical component of protein kinase A signaling^43^, heterodimerizing with Creb1 to modulate gene expression in vertebrates. Indeed, according to our data, both Atf3 and Creb1 appear to be involved in instituting or maintaining the foraging state (Table 3), suggesting that they might be acting in a cooperative manner in bees as well. Usf1, by contrast, is known to work in opposition to Creb1 signaling^44^ and is similarly predicted by our data to repress forager-related transcripts, potentially countering Creb1’s predicted role as an activator of foraging related genes. Several modulators of NF-κB activity, including *egr*^45^ (another putative regulator detailed below) and *nr4a2* (which interacts with NF-κB in the nervous system^46^) were also found to be differentially expressed in foragers. Together, these groups of genes may represent coherent regulatory modules governing behavioral state. At the very least, the fact that so many TFs known to interact with one another are predicted to regulate the same behavioral state reinforces the idea that the cellular functions regulated by Creb1 and NF-κB are particularly vital for the onset or maintenance of foraging behavior.

EGR, a TF that has previously been characterized as a canonical immediate early gene (IEG) linked to induction of neural plasticity in a variety of organisms^47^, was also upregulated in foragers. *egr* expression in the honey bee mushroom bodies (a region of the insect brain involved in learning and memory) is responsive to stimuli that trigger spatial learning (namely orientation flight) in conjunction with exposure to a novel environment^48^. Quantitative PCR analyses additionally indicate that mushroom body *egr* expression increases in association with behavioral maturation independent of environmental stimuli^48^. Our results concerning *egr* are therefore consistent with previous findings. Moreover, since the *egr* motif is enriched in the promoters of forager up-regulated genes, these data suggest that *egr* functions not only as an IEG that governs transcriptomic responses to experiential stimuli, but also helps orchestrate the neurotranscriptomic changes that precede and maintain the foraging state as well.

The gene *rxra1* (*ultraspiracle/usp*) is a highly conserved nuclear receptor with affinity for both juvenile hormone^49^ and ecdysone. Its identification as a putative regulator of foraging behavior is fitting, since endocrine signals (including juvenile hormone) are known to play a critical role in regulating behavioral maturation in honey bees^50^. Moreover, experimental *usp* knockdown was previously shown to delay the transition to the foraging state^36^. This indicates that CAGEscan can “reproduce” known causal effects of genes on behavioral state, something that approaches based purely on informatics-derived inferences have sometimes failed to capture^5^. Intriguingly, the gene for Ecdysone Receptor (*EcR*), a binding partner of USP^51^, was also upregulated in foragers. While ecdysone has no known role in honey bee behavioral maturation, the co-expression of *ecdysone receptor* with its binding partner *usp* provides a suggestive hint that such a relationship exists, but has hitherto gone undetected.

The identification of *clockwork orange* (*cwo*), a critical component of the circadian regulatory circuit in *Drosophila melanogaster*^52^, as a putative regulator of behavioral maturation is also noteworthy. Although adult honey bees appear to possess endogenous biological rhythmicity from the moment they emerge from their cells, their locomotor behavior and metabolism are largely arrhythmic until shortly before the onset of foraging^53^. Correspondingly, circadian related gene expression begins at a low and relatively invariant level, gradually increasing and becoming rhythmic as the bee approaches the foraging state. Additionally, the ability to form time-dependent memory is critical for honey bees, since they forage on resources that are both spatially and temporally restricted. Not only must a forager remember where a previously visited floral patch is, it must know when a floral patch is producing nectar and pollen. A previous study assaying brain gene expression changes in foragers found that the expression of genes associated with circadian rhythmicity not only cycle as a result of the time of day, but can also be modulated by training a bee to forage at a particular time point, suggesting they play a critical, perhaps even causal, role in organizing the temporal aspects of a bee’s foraging behavior^34^. Since all nurse and forager samples were collected within a very short time window (less than 1.5 hours), variation in *cwo* levels due to time of day should be minimal, suggesting that this gene may instead be serving a crucial function in the onset of spatiotemporal learning in honey bee foragers.

Finally, several TFs associated with nervous system development in *Drosophila* were also identified as putative regulators of the foraging state, namely: *hes1* (in flies known as *hairy* or *deadpan*), *dri* (*retained*), *pax6* (*eyeless*), *hoxA6* (*deformed*), and *hoxA1* (*labial*). Additionally, motifs associated with two of these TFs (*dri* and *hairy*) have previously been identified as enriched in the promoter regions of genes that are associated with behavioral maturation^37^. Neural plasticity associated with behavioral maturation in honey bees is known to involve large increases in dendritic arborization in specific brain regions^54^, and the cooption of developmental transcriptional programs may be one way this plasticity is mediated.

Remarkably, motifs associated with five differentially expressed transcription factors (*hes1* (*hairy*), *pax6*, *NF-κB*, *egr* and *clockwork orange*) were combinatorially enriched in the promoters of nearly 50% of the genes that were upregulated in foragers (Supplementary Figs S8 and S9, Supplementary Dataset 7). This suggests that a large proportion of the brain transcriptomic differences between nurses and foragers may be influenced by a small number of TFs, a pattern that also has been predicted by previous bioinformatic analyses^5^. Moreover, the fact that such a large number of motifs were enriched in the same set of promoters implies that these five genes may co-regulate a coherent module of the regulatory architecture underlying behavioral state. It should be noted that the motif associated with one of these TFs (*pax6*) did not have the same level of enrichment when all OGS v3.2 promoters were used as the set of background sequences, suggesting that G/C bias may have had an influence in the detection of this particular motif (Table 3). Regardless, even if only the other four TFs are considered as putative co-regulators of such a significant proportion of the forager transcriptome, this is still a remarkable finding.

By contrast, only a single differentially expressed transcription factor, *MyoD* (*nautilus*), was associated with a motif enriched in more than 50% of the nurse upregulated genes. Traditionally known as a master regulator of cell fate in muscle cells^55^, *MyoD* has only recently been characterized in the nervous system, where its only known function is as a tumor suppressor in the cerebellum of vertebrates^56^. As such, this is the first discovery of the potential involvement of a MyoD ortholog as a key regulator of behavioral state, and elucidating its role in the insect nervous system will require additional study.

### Alternative Transcriptional Start Sites and Behavioral Maturation

**Figure 7:**
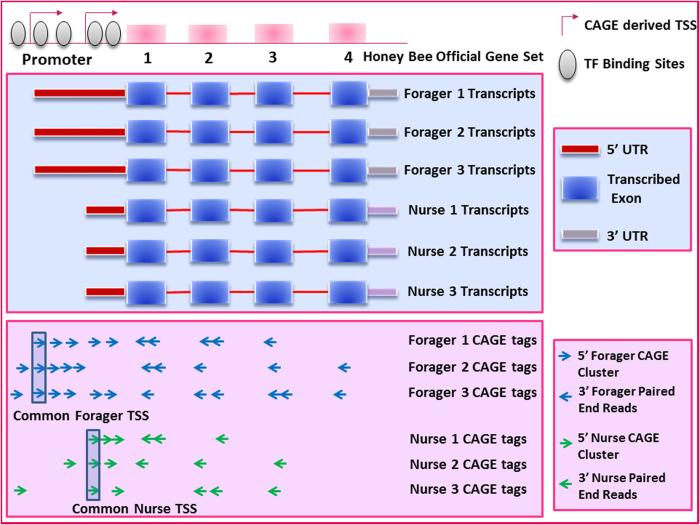
CAGE tags mapping, clustering and TSS identification. After sequencing, the CAGE tags were mapped to v3.2 of the honey bee Official Gene Set to form clusters (the 3’paired end reads are used to facilitate this mapping). A cluster was identified as a ‘common’ TSS for each group if it had the greatest number of CAGE tags relative to all other clusters and was present in all samples within the group. The location of the forager and nurse TSSs was then compared to determine whether differential TSS selection occurred as a consequence of behavioral state. Additionally, promoter regions identified using CAGE can be scanned for differences in TF binding site occurrence to gain insights into the regulatory architecture controlling each gene’s transcription.

In order to determine whether alternative TSSs were associated with behavioral state, we analyzed their occurrence in nurse and forager upregulated genes. For our purpose, TSSs are defined as the CAGE cluster with the highest degree of coverage (i.e., the most transcribed) that is common to all samples within a group (Fig. 7). We first identified genes with multiple CAGE clusters across samples (Supplementary Fig. S10), and then compared the results of our TSS analysis at each of these loci to determine whether there were systematic differences in TSS usage between foragers and nurses. Differential TSS usage was defined as the existence of distinct common TSSs in nurse and forager samples separated by a mutual distance of at least 100 bp (Fig. 7).

Our data indicate that 646 out of the 12,453 expressed genes possessed alternative TSSs that were utilized differentially between nurses and foragers (Supplementary Dataset 8). However, only 14.9% (96/646) of these genes were also found to be differentially expressed between nurses and foragers (Supplementary Dataset 8). One potential interpretation of this small proportion is that, if alternative TSS selection plays a substantial role in regulating behavioral maturation in the honey bee, it does so by mediating splicing or post-transcriptional regulation of the resulting transcripts rather than directly influencing the levels of transcript produced. Alternative TSSs have been shown to have a significant effect on isoform expression (through differential recruitment of splicing factors or the exclusion of 5’ exons)^57^, mRNA turnover, and the efficiency of translation^58^ in other species, so it is reasonable to speculate that they serve a variety of similar functions in the honey bee as well. Still, although the overlap between DEGs and alternative TSSs is small, the prevalence of genes with alternative TSSs is significantly higher in DEGs than in the whole transcriptome (Fisher's right-hand exact test using hypergeometric distribution. p-value < 3e-08). As such, it’s still possible that alternate TSSs play at least some role in regulating the rate of transcription during behavioral maturation.

Additionally, the small number of identified alternative TSSs relative to previous studies is related, in part, to our use of highly stringent criteria for the identification of TSSs. While the beginning of each CAGE tag can be considered as a discrete TSS, clustering CAGE tags is necessary to avoid false TSSs^59^. Moreover, since we were interested in delineating the systematic differences in TSS between nurses and foragers, we clustered all CAGE tags within a 50 bp window to determine a consensus start site for each group of bees. This provided a much more coherent picture of the distinct trends in start site selection between these two groups. In order to prevent tags from overlapping consensus sites, we further required that each alternative start site be separated by a mutual distance of at least 100 bp. Relaxing either of these constraints dramatically increases the number of genes exhibiting alternative TSSs (Supplementary Figure S11). One should therefore consider the 646 genes with alternative TSSs to be a very conservative estimate of the link between behavioral state and TSS selection in the bee. Regardless, these results provide the first evidence that alternative TSSs reflect transcriptomic changes that are associated with sustained differences in behavior.

Gene Ontology (GO) analysis of the 646 genes with alternative TSSs show enrichment for a set of GO terms associated with nervous system development, neuronal development, axon guidance, wing development, oxidoreductase activity, lipid biosynthesis process and respiratory system development (Supplementary Dataset 9). These terms are strikingly similar to those obtained by GO analyses of DEGs (despite the low prevalence of DEGs exhibiting differential TSS usage) and are strongly suggestive of a role for alternative TSS usage in establishing and/or maintaining differences in nervous system function between nurses and foragers.

## Conclusions

For the first time, we experimentally determined the TSSs and transcribed promoter regions associated with the regulation of behavioral plasticity in bees. We showed that the promoters of DEGs are enriched for motifs associated with many of the TFs we found to be differentially expressed, highlighting the potential importance of these TFs in regulating behavior. The coherent picture presented by our data and previous experimental and bioinformatics results reveals that CAGEscan provided us with highly detailed and convincing evidence about the functional architecture underlying the transcriptome during behavioral maturation. For instance, a number of these TFs were previously predicted to regulate behavioral maturation, and nearly all of them are associated with functions that correspond to known aspects of behavioral maturation.

Additionally, we found that a small subset of these putative regulators of behavioral state might be responsible for organizing the majority of transcriptomic differences in nurses and foragers, a result that corresponds with previous regulatory network analyses^5^. These results contribute to a growing appreciation of the fact that many behavioral states are associated with (and presumably regulated by) extensive and distinct transcriptional signatures in the brain^3^,^60^. However, how such changes in RNA abundance lead to changes in neuronal function and, subsequently, behavior is a challenge that remains to be solved.

The fact that motif enrichment was assessed in actively transcribed promoter regions makes it all the more likely that the enriched motifs serve a functionally relevant role^12^ in the transcriptional regulation of behavioral state. This is supported by the number of putative regulators that have previously been implicated in controlling behavioral maturation (Table 4). Still, we must stress that our results are purely correlative. Future studies should attempt to assess the veracity of these predictions by making targeted manipulations of these TFs and ascertaining their effect on behavioral state and the expression of predicted target genes.

Additionally, while the ability to associate differential TF and target gene expression with motif enrichment in actively transcribed regions is strongly suggestive of regulatory function, one should not expect all of a transcription factor’s potential targets to be regulated in every context, particularly since genes are not commonly under the control of a single TF. Therefore, additional experiments are required to study how the combinatorial interactions between these TFs affect the expression of each target gene and give rise to contextually specific patterns of gene expression. Our findings implicate five TFs as putative co-regulators in nearly half the genes that were upregulated in foragers, which implies that TF co-association at the promoter may play a vital role in instituting or maintaining behavioral state. Because such combinatorial interactions have previously been predicted to play important roles in governing behavioral maturation^6^ and TF co-association at the promoter appears to drive evolutionarily conserved differences in contextually dependent gene expression during development^13^, dissecting these patterns of co-regulation using targeted manipulations of the putative regulators is a logical next step in elucidating how the brain transcriptome organizes behavior.

Similarly, the lack of motif enrichment for differentially expressed TFs should not be construed as evidence that they are not involved in the regulation of behavioral maturation, particularly since the assay used here cannot account for the potential presence of TF binding sites at enhancers distal to the gene promoter. Similarly, this limitation makes it likely that a significant number of real targets were not characterized by CAGEscan. Therefore, the analyses presented here should be used to motivate and inform future experiments to study physical occupancy of potential binding sites by the most promising TFs, as has been done previously for Ultraspiracle Protein^36^.

It should be noted that, unlike previous studies^30^,^31^, we did not control for the effect of age on gene expression. Since the transition from hive-bound to foraging tasks involves a developmental trajectory, this presents a potential confound for our findings. However, previous studies assessing the contribution of chronological age relative to other maturational determinants have found that age plays a relatively minor role in determining differences between nurse and forager brain transcriptomes^30^. Moreover, age-related differences in brain gene expression are most apparent in early adult life, generally long prior to onset of nursing and foraging behavior^30^. Our results also exhibit high concordance with the predictions of a meta-analysis that assessed the link between maturational determinants (other than age) and transcriptomic architecture^36^. As such, we feel that while the potential age differential between nurses and foragers is doubtless responsible for some alterations in gene expression, it is unlikely to affect our overall conclusions.

Finally, applying CAGEscan we were able to identify reliable differences in TSS selection related to behavioral state for the first time. The transcripts for a substantial number of genes exhibit start sites unique to nursing or foraging behavior, and GO analysis indicates that these are relevant to nervous system function. While alternative TSSs may be regulating transcriptional rates in a comparatively small proportion of these genes, it’s also possible that they are contributing to the expression of alternative isoforms or other post-transcriptional regulatory processes that may contribute to the regulation of behavioral plasticity.

## Methods

### Sample Collection

All samples were collected from a single colony at the University of Illinois Bee Research Facility, Urbana, Illinois. Samples were the offspring of a queen inseminated with semen from a single drone, which (due to the haplodiploid genetics of the honey bee) results in worker offspring with 75% average genetic relatedness. Behavioral identification was according to standard methods^36^. Bees that were observed entering honeycomb cells containing larvae were identified as nurses, immediately collected using forceps, and frozen in liquid nitrogen. Bees returning to the colony with loads of pollen on their hind legs were identified as foragers, captured using soft forceps and immediately frozen in liquid nitrogen. All collections (N = 25 nurses and foragers) were performed within a 1.5 hour timespan (from 10:00 to 11:30 a.m.) on the same day (July 29^th^, 2011). After collection, bee heads were freeze dried and brains were dissected in 80% ethanol chilled using dry ice^61^.

### RNA extraction

Total RNA from individual bee heads was prepared by homogenizing the brain tissue using a motorized pestle and extracting the RNA using TRIzol (Life Technologies, Carlsbad, California, USA) and RNeasy Mini spin columns (Qiaqen, Venlo, Limberg, Netherlands), as per manufacturer specifications. All samples were treated with DNase (Qiagen). Sample quality was confirmed using a Nanodrop (Thermo Scientific, Walthan, Massachusettes, USA) and Bioanalyzer (Agilent Technologies, Santa Clara, California, USA).

### CAGEscan Library Construction

CAGEscan libraries were generated from total RNA preparations of individual bee brains (16 samples including 8 nurses and 8 foragers), and the barcoded cDNAs were pooled into two libraries for sequencing using established protocol^62^ (Fig. 1). This protocol was modified slightly to reduce the rRNA content of CAGEscan libraries and to improve the selection of true 5’ ends by incubating the RNA in 5’-Phosphate-Dependent Exonuclease (Terminator, Epicentre, Madison, USA) to remove rRNA and truncated mRNAs. During cDNA synthesis a reverse-transcription primer and “template-switching” oligonucleotide with individual barcodes (Supplementary Table S8) plus specific sequences for template switching at the 5’ cap of mRNA were incorporated into the first strand cDNA by a reverse transcriptase. Since the primer and template-switching oligonucleotide added known sequences to the 5’ and 3’ ends of the cDNA, they could be used as templates for semi-suppressive PCR. Using this process, long strands of cDNA were selectively amplified to generate the second cDNA strand (molecules that were short or possessed the same adaptor sequences at their 5’ and 3’ ends self-hybridized prior to the PCR, precluding amplification). The length of cDNA fragments within the CAGEscan library preparations ranged from 200–700 bps.

### Sequencing of CAGEscan Libraries

Sequencing of CAGEscan libraries was performed by the W.M. Keck Center for Comparative and Functional Genomics (University of Illinois at Urbana-Champaign, Urbana, Illinois, USA). Nurse and forager samples were combined into separate pools, and sequenced in different lanes and sequencing runs. Upon sequencing the forager samples, the quantity of reads obtained was judged to be lower than desired, and additional input cDNA was used for the nurse samples. CAGEscan tags used in this study were paired-end reads of length 100 bp. Low quality and outlier reads were filtered out of the data sets using FASTQC (http://www.bioinformatics.bbsrc.ac.uk/projects/fastqc), and CAGE tags with missing or incorrect adapters were omitted. In the sequence trimming process we removed the adapter sequence (21 bp, Supplementary Table S8) from the first mate of the paired-end sequences, and correspondingly pruned part the second mate, such that both mates had equal lengths (79 bps).

### Mapping and Filtering CAGE Tags

The 79 bp paired-end reads obtained after trimming were aligned to the honey bee reference genome (version 4.5) using Bowtie2 v2.1.0^63^ in order to calculate the estimated mean (588 bp) and standard deviation (767 bp) of the inner distance between mapped paired-end reads. These parameters were then used with the Tophat v2.0.8^64^ splice junction mapper to improve our ability to align the reads to the reference genome, allowing for up to 2 mismatches and 2 gaps per read. For the CAGE tag filtering process, we filtered out mapped reads that had a relatively high probability (p > 0.01/MAPQ < 20) of being mapped incorrectly. Paired reads also were excluded from further analyses when: 1) both mates mapped to alternate strands, 2) one mate was unmapped, 3) the mates mapped to different scaffolds or 4) there was an inner distance greater than (mean + standard deviation) of the estimated inner distance between paired reads.

### Gene Expression

The CAGE tags were mapped to the official honey bee gene set, OGSv3.2^32^. A typical CAGE tag was considered to be associated with a gene if it intersected with the region that covers [-2000 bp, end of the gene], but may be restricted by the end of the upstream gene on the same strand. In these cases the tag was considered to be associated if it maps to the region [end of the upstream gene + 1, end of the gene]. As such, it is possible for multiple CAGE tags to be associated with one gene, or one CAGE tag to span two adjacent genes. To insure that the mapped reads provided sufficient coverage for differential expression analyses, their distribution was plotted using RSeQC^65^. We generated a gene expression data matrix using the association of tags and genes, where each row represents the expression levels for a gene and each column represents a nurse or forager samples. Only those genes that had non-zero expression level in at least two samples of any of the nurse/forager groups were used for downstream analyses. Using this matrix, we normalized gene expression by rescaling the number of tags from each sample to the minimum number of tags from across all samples to remove sequencing bias.

### Gene Clustering Based on Expression

To determine the differences in brain gene expression levels between nurses and foragers, we performed two-way unsupervised hierarchical clustering using MATLAB to cluster genes and samples using an inner squared distance (minimum variance) algorithm. The Euclidean distance metric was used to measure the distances between gene profiles (rows) and Pearson’s correlation coefficient was used to measure the distance between sample profiles (columns). To obtain a statistical measure of how the clustering preserves the actual dissimilarities between samples, an unsupervised evaluation of hierarchical clustering using cophenetic correlation coefficient (CPCC) was performed. The CPCC is defined as:


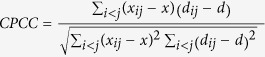


where *x*_*ij*_ is the Euclidean distance between *i*^*th*^ and *j*^*th*^ observation and *d*_*ij*_ is the cophenetic distance, which is the height of the link that joins the two observations in the obtained clustering dendrogram; *x* and *d* are the averages of *x*_*ij*_ and *d*_*ij*_, respectively. CPCC is the linear correlation coefficient between the observed distances (dissimilarities) in the samples and the cophenetic distances obtained from the clustering. In our case the CPCC was 0.78, suggesting that the clustering was not a technical artifact but represents actual biological differences between samples.

### Variability of Gene Expression

We evaluated differences in brain gene expression between individual bees within the nurse and forager groups by calculating the per-gene variance in expression levels between the individuals within each group. The variance was calculated on scaled expression data using the Z-score, such that the expression values of each gene had a mean equal to zero and standard deviation equal to 1. To examine if the variation in gene expression between forager samples was significantly different from the variation between nurse samples, we used the Wilcoxon Rank-Sum test between the two vectors of variances. Finally, we compared the samples using the per sample biological coefficient of variation (the square root of the dispersion parameter for the 500 most variable genes) and the per gene squared coefficient of variation (CV^2^) (the squared ratio of the standard deviation of gene expression across all group samples to the group average gene expression).

### TSS Identification, Differential TSS Usage and Promoter Extraction

To define TSS positions, CAGE tags belonging to each sample were clustered using an iterative hierarchical clustering approach with Paraclu v9^66^ to form clusters covering regions of less than 50 bp (Fig. 7). Clusters that were more than 50 bp in length or were represented by fewer than 5 tags after rescaling were removed. Clusters with a maximum density/baseline density ratio of less than 2 also were excluded (since the signal strength was likely insufficient to represent a real TSS), as were clusters that were merely components of a larger cluster. We used these CAGE clusters to identify potential gene TSSs for the nurse and the forager groups independently of one another. Because more than one CAGE cluster could potentially be associated to a particular gene, we defined a gene’s TSS to be the starting position of the CAGE cluster that has the greatest overall number of CAGE tags and is present in all of the samples in a group. These sites therefore represent a set of common TSSs for the expressed genes in each of the groups. To determine whether there was differential TSS usage between nurses and foragers, we compared the common TSS for each group. Those genes with distinct TSSs for each group were judged to use alternative start sites as a consequence of behavioral state. Due to the potential overlap of paired end reads in adjacent CAGE clusters, only TSSs with a mutual distance >100 bp were considered for this analysis.

Promoters were defined as regions covering [-2000 bp, 200 bp] relative to TSSs common within a group. The final promoter region was further constrained so that it did not overlap an upstream gene or exceed the stop codon of the downstream gene to which the promoter was associated. Despite this restriction on promoter length, 65% of all OGSv3.2 genes (and 75% of differentially expressed genes) still use the full promoter region. Only 19% of OGSv3.2 genes (and 13% of differentially expressed genes) have promoters of <1000 bp length, and 9% of OGSv3.2 genes (and 5% of differentially expressed genes) have promoters of <500 bp length.

### Differentially Expressed Genes (DEGs)

The brain gene expression profiles of eight nurses and eight foragers were determined from the raw count of the CAGE tags associated with the respective genes. We filtered genes with a low level of expression, keeping only those that had at least 1 tag per million reads in at least 2 samples. To remove sequencing bias due to coverage depth, gene expression data were normalized using the Trimmed Mean of M-values (TMM) method^67^. Differentially expressed genes were determined on a per gene basis. We always compared genes of the same length to find differences in expression between the samples of each group, and gene length had no influence on the results. This allowed us to normalize based purely on the distribution of reads across the genes using the TMM in edgeR^68^. Statistical analyses of gene expression data to identify DEGs were performed in edgeR using tagwise dispersion to estimate the variance within each gene. EdgeR’s implementation of Fisher’s Exact Test (which corrects for overdispersion and uses a negative binomial distribution) was then performed to evaluate differential expression, and the resulting p-values were adjusted for multiple comparison testing using the Benjamini-Hochberg false discovery rate (FDR < 0.05).

The honey bee brain is surrounded by a large exocrine organ called the hypopharyngeal gland (HPG), which presents a potential source of contamination. Moreover, the HPG’s size and level of activity varies substantially in nurses and foragers, making it possible for contamination to bias gene expression assays and increase Type I error. Since it is impossible to quantify potential contamination directly, previous studies of nurse-forager gene expression have excluded genes with a high level of expression in the HPG^31^. To determine whether this would be necessary for our data, we used RNAseq to quantify the expression of genes in the HPGs (relative to brain tissue) of nurses and foragers. The top 1%, 5%, 10%, and 20% (by log fold change) of genes upregulated in the HPG of each group were then compared to their respective CAGEscan DEGs to determine the level of overlap. Since contamination is far more likely in nurse samples, genes that were upregulated in forager HPGs but also in the top 10% of nurse HPG upregulated genes were excluded from the forager overlap analysis (if contamination had occurred, it would have resulted in the false identification of nurse, rather than forager, upregulated genes).

### DEG Overlap with Previous Studies

To demonstrate the validity of data derived from CAGEscan and to provide a coherent picture of the genes that are most consistently differentially expressed in the brain as a function of behavioral maturation, we compared our results with those reported in two previous studies^29^,^31^. Previous studies were performed using two independently designed microarrays: one^29^ containing ~9,000 probes based on honey bee expressed sequence tag data that predated the sequencing of the honey bee genome (Array Express Accession #A-MEXP-36), and a second^31^ with ~13,000 probes derived from gene annotations (OGS 2.0) for Assembly 2.0 of the sequenced genome (Array Express Accession #A-MEXP-755). For consistency, these datasets were reanalyzed by mapping the microarray probes to the current official honey bee gene set, OGSv3.2^32^ using BLAT and Bowtie. Probes that could not be mapped to a unique gene were not used for further analyses. The microarray data were then corrected for multiple comparisons using a FDR cutoff of 0.05. In instances where multiple differentially expressed probes mapped to the same gene, the probes invariably exhibited the same direction of expression change across experimental groups. Therefore, duplicate probes were ignored. The significance of the overlap between each gene list was calculated using hypergeometric tests in SAS v9.4 and adjusted for multiple comparisons using a Bonferroni *post hoc* correction.

### Functional Annotation of DEGs

Gene Ontology (GO)^69^ terms for the DEGs were determined using orthology to the *Drosophila melanogaster* genome, resulting in a total of 4,999 GO terms. GO enrichment analysis was performed based on the frequency of terms associated with the forager/nurse DEG list relative to the genomic background (all genes that had detectable levels of expression) using Fisher’s exact test, followed by FDR correction for multiple testing (FDR < 0.05). Analyses were performed using DAVID^70^ and the category-frequency of enriched GOs was analyzed using CateGOrizer^71^.

### Identification of TFs and Motif Finding around TSSs

After analyzing differential expression, we identified TFs with Position Weight Matrix (PWM) models available in other organisms. To do so, we composed a list of 1,402 TFs (and their isoforms) associated with 676 PWMs from three different sources. We used 1,000 Human TFs from HOCOMOCO v9 database^72^ associated with 426 PWMs, 217 *Drosophila* TFs from Flybase^73^ associated with 73 PWMs, and 185 insect TFs from TRANSFAC Professional ver. 2012.2^74^ associated with 177 PWMs (Table 5). Then we compared the protein sequences of these TFs to the 15,314 protein sequences of *A. mellifera* OGS3.2 using OrthoMCL^75^ to find orthologous TFs. To identify TFs that might be key regulators of the nursing and foraging behavioral states, we used Clover^76^ to assess whether associated motifs were overrepresented in the promoters of genes that were upregulated in nurses and foragers; motifs with similarity scores greater than 6 and a significance level of p-value < 0.05 were considered to be enriched. TFs that were differentially expressed and were associated with motifs enriched in genes upregulated in nurses or foragers were considered to be putative regulators of those respective behavioral states.

A previous informatics analysis uncovered a systematic bias toward high Guanine/Cytosine (G/C) content in the promoters of genes upregulated in foragers (relative to nurse associated promoters)that led to an overestimate of the number of overrepresented TF motifs associated with behavioral state^37^. To ascertain whether a similar bias exists in the CAGE tags that comprise our dataset, we compared the ratio of G/C to A/T nucleotides in the reconstructed promoters of forager and nurse upregulated genes using the Wilcoxon Rank-Sum Test. We then accounted for differences in G/C content by performing our *cis*-motif enrichment analysis using three different backgrounds. For the first test, the background consisted of promoters from genes upregulated in the behavioral state that was not being assessed (i.e., the promoters of forager upregulated genes used the promoters of nurse upregulated genes as a background) in order to emphasize the distinctions in motif distribution between these sets of promoters. We then performed two additional analyses to confirm the validity of these findings, using either: 1) all predicted promoters in OGS v3.2 or 2) randomized portions of the bee genome as the background for each set of promoters.

## Additional Information

**How to cite this article**: Khamis, A. M. *et al.* Insights into the Transcriptional Architecture of Behavioral Plasticity in the Honey Bee Apis mellifera. *Sci. Rep.* **5**, 11136; doi: 10.1038/srep11136 (2015).

**Accession codes**: CAGEscan Sequences for nurse and forager samples have been deposited in NCBI GEO under the accession number [GSE64315].

## Supplementary Material

Supplementary Information

Supplementary Dataset 1

Supplementary Dataset 2

Supplementary Dataset 3

Supplementary Dataset 4

Supplementary Dataset 5

Supplementary Dataset 6

Supplementary Dataset 7

Supplementary Dataset 8

Supplementary Dataset 9

## Figures and Tables

**Table 1:** Number of genes that could be associated with CAGE tags.

	**Number of genes**
Total number of genes in OGS3.2	15,314
Number of genes associated with CAGE tags in at least one sample	13,111 (85.6%)
Number of genes associated with CAGE tags in two or more samples	12,453 (81.3%)

**Table 2:** Putative Transcriptional Regulators of Behavioral State.

	**Human Ortholog**	**Fly Ortholog**	**Bee Gene Identifier**
	USF1/USF2	*usf*	GB40634
Nursing Regulators	CXXC1	*cfp1*	GB43820
	MYOD1	*nautilus*	GB55306
Foraging Regulators	RXRA/RXRB	*ultraspiracle*	GB42692
	CREB1	*Creb-B17A*	GB46492
	C/EBP	*slbo*	GB44204
	DFD	*deformed*	GB51299
	HXA1	*labial*	GB51303
	ATF3	*atf3*	GB53401
	DRI	*retained*	GB55596
	NF-κB	*dorsal*	GB42472
	EGR1/EGR2	*stripe*	GB50091
	PAX6	*eyeless*	GB50342
	HES1	*Hairy*	GB47799
	BHE40/BHE41	*cwo*	GB52039

**Table 3:**
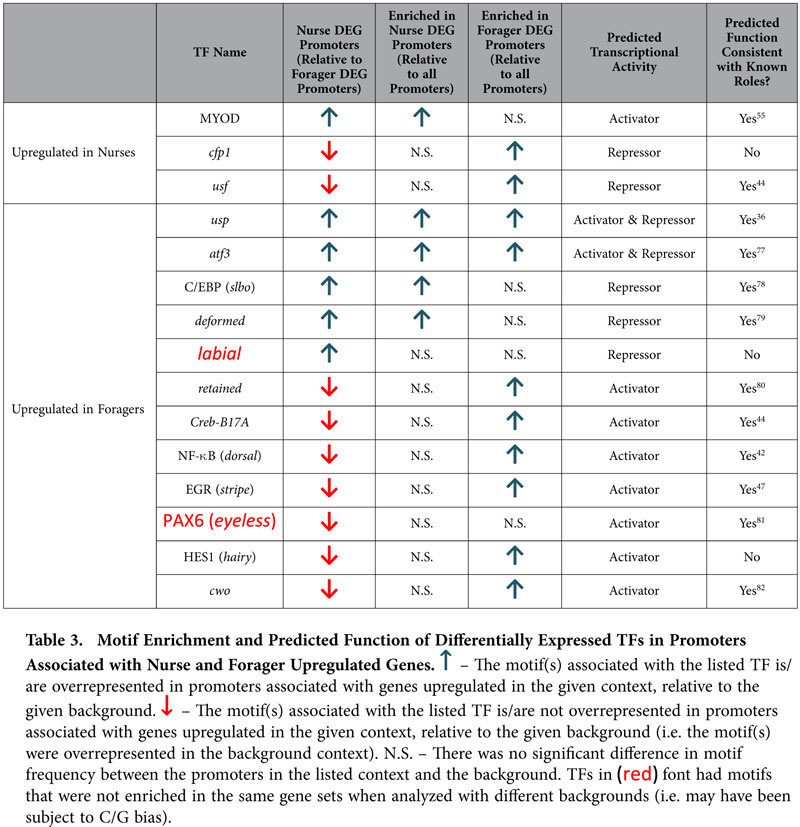
Motif Enrichment and Predicted Function of Differentially Expressed TFs in Promoters Associated with Nurse and Forager Upregulated Genes.

**Table 4:**
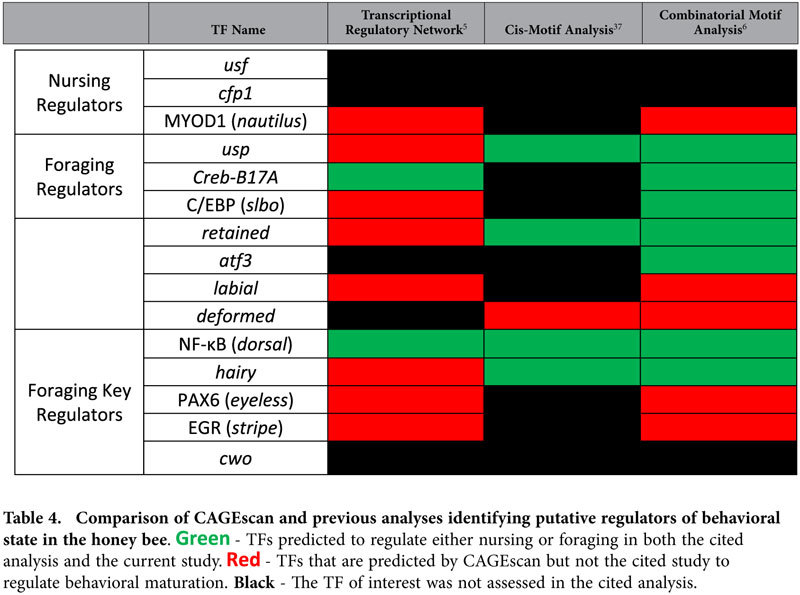
Comparison of CAGEscan and previous analyses identifying putative regulators of behavioral state in the honey bee.

**Table 5:** Summary of the 676 PWMs associated with 1,402 TFs collected from TRANSFAC, Flybase and HOCOMOCO.

	**Number of PWMs**	**Number of TFs Without Isoforms**	**Number of TFs and Isoforms**
TRANSFAC	177	133	185
Flybase	73	142	217
HOCOMOCO	426	405	1,000
Total	676	680	1,402

**Figure:**
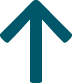


**Figure:**
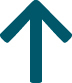


**Figure:**
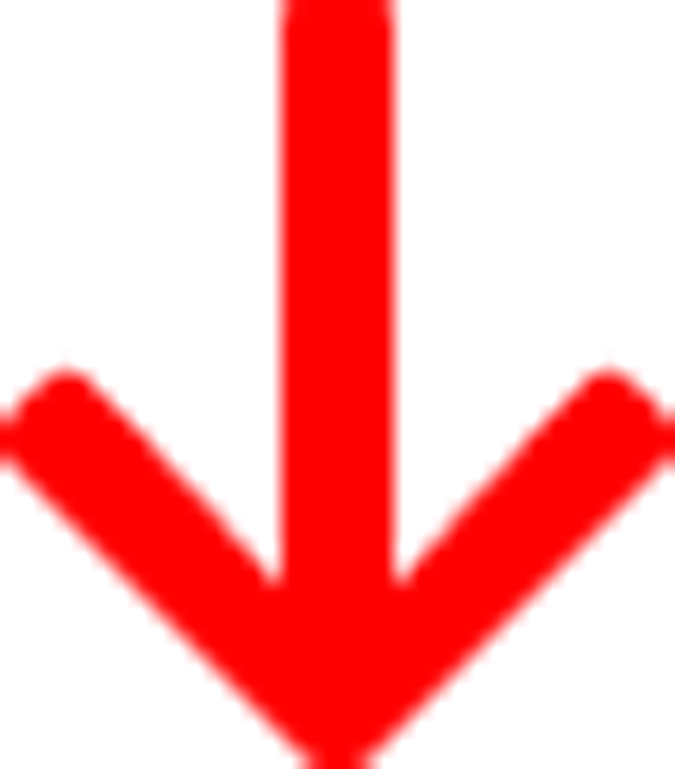


**Figure:**
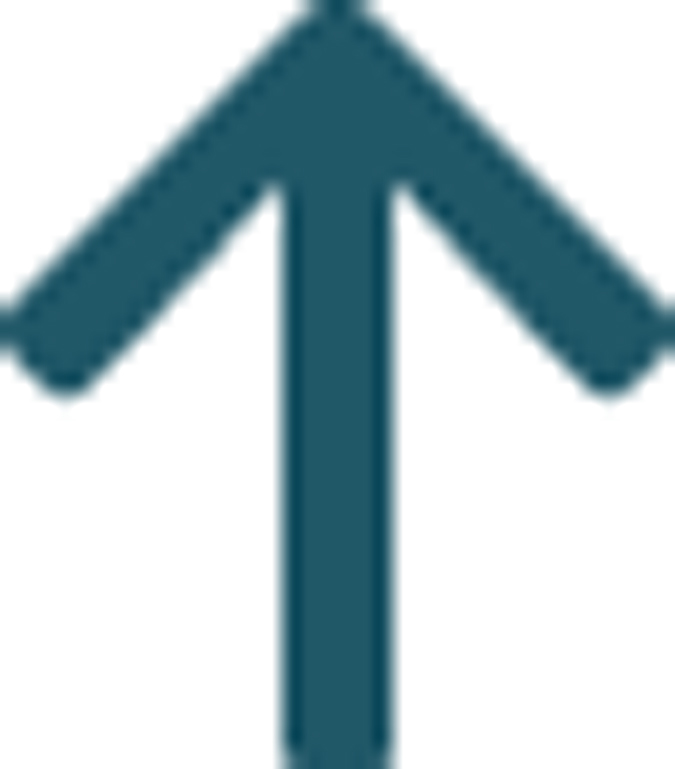


**Figure:**