# Supplementary material for: Insights into the Transcriptional Architecture of Behavioral Plasticity in the Honey Bee Apis mellifera
Source: Sci Rep. 2015 Jun 15;5:11136. doi: 10.1038/srep11136 (PMC4466890; doi:10.1038/srep11136)
Supplement: Supplementary Information [file srep11136-s1.pdf]

Supplementary Materials

for

"Insights into the Transcriptional Architecture of Behavioral Plasticity in the Honey Bee *Apis mellifera*"

Abdullah M Khamis[1*], Adam R Hamilton[2*], Yulia A Medvedeva [1&], Tanvir Alam[1], Intikhab Alam[1], Magbubah Essack[1], Boris Umylny[3], Boris R. Jankovic[1], Nicholas L. Naeger[2], Makoto Suzuki[4], Matthias Harbers[4,5#], Gene E. Robinson[2#], Vladimir B Bajic [1#]

Supplementary Figures

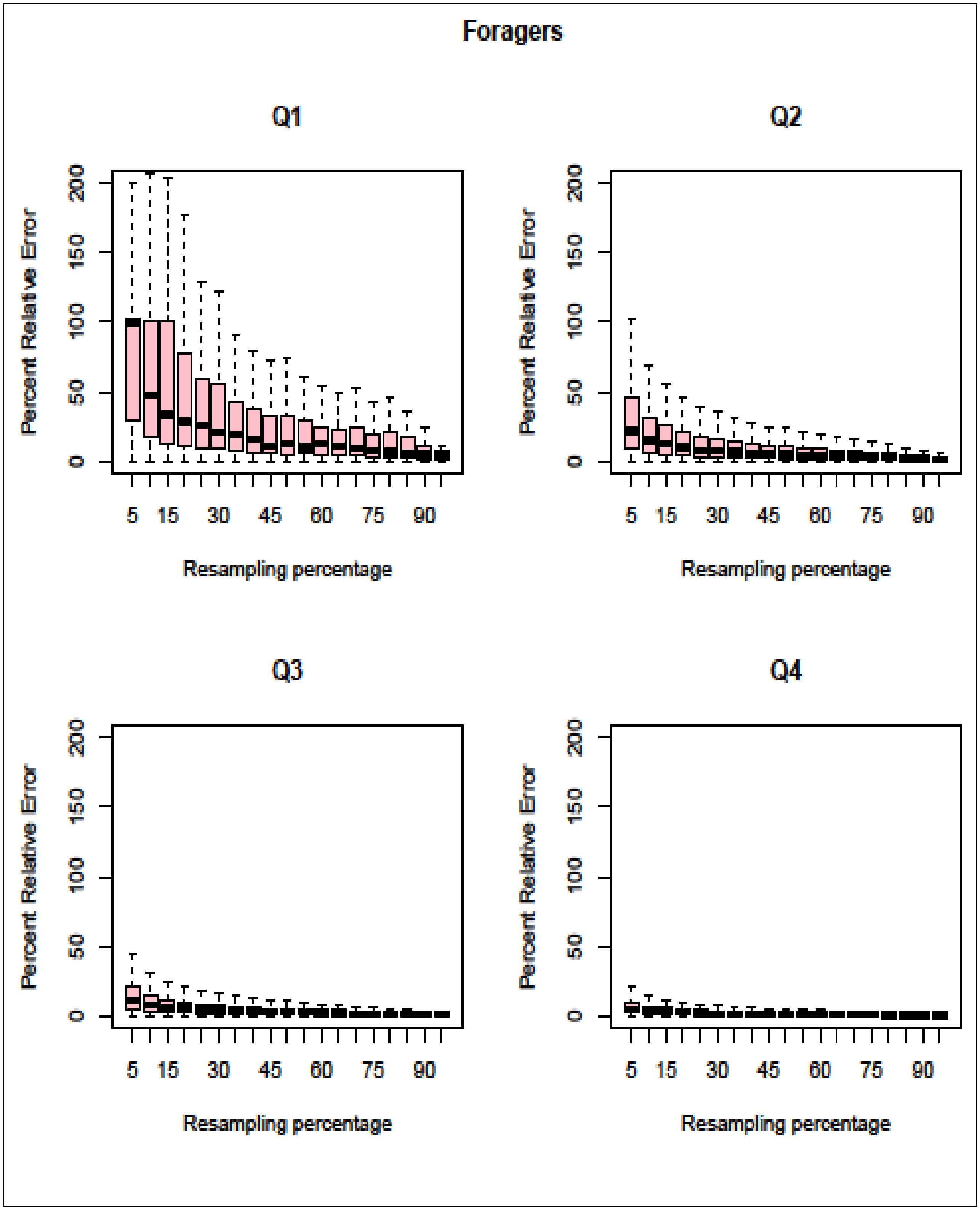

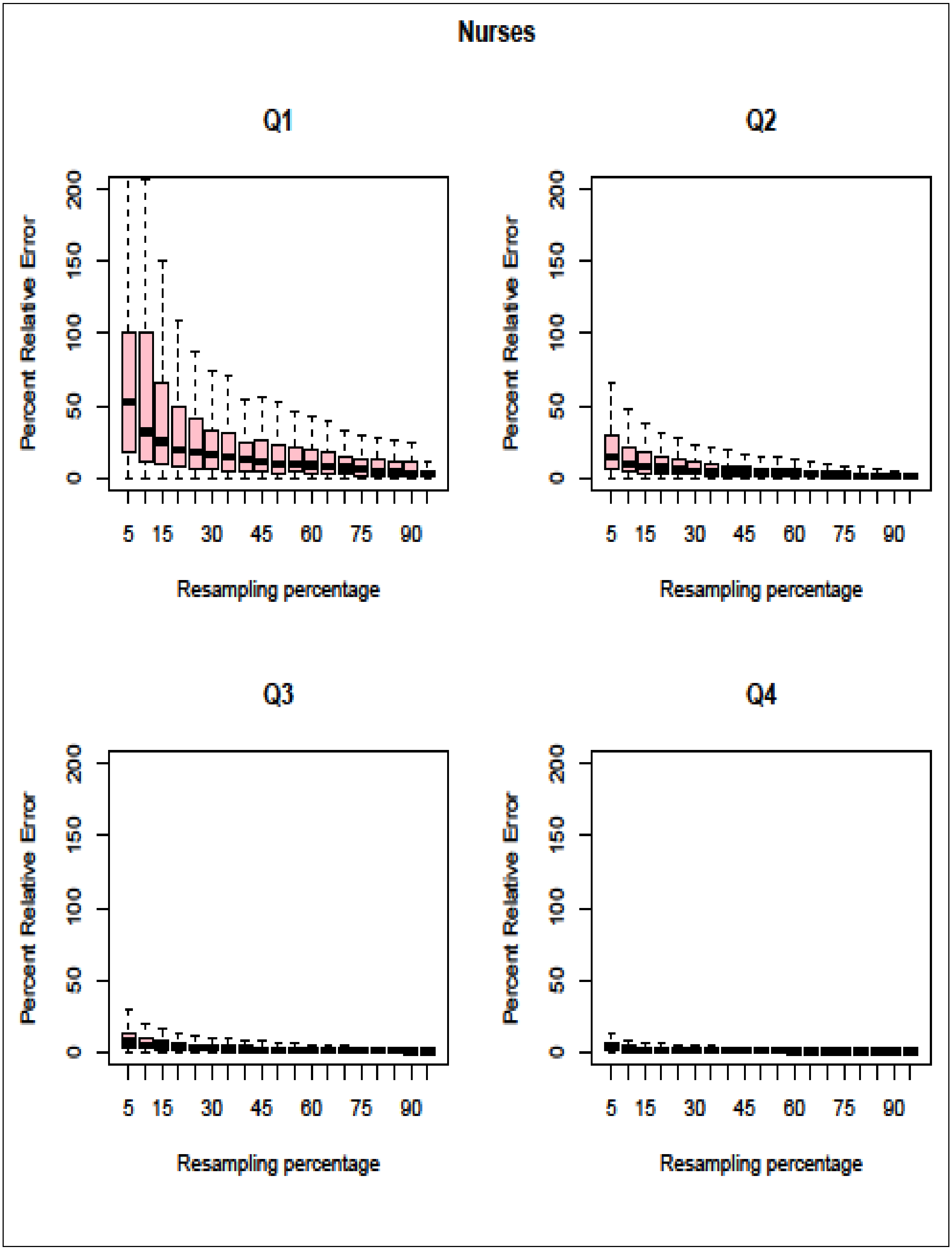

Nurses
Q1
Q2
Q3
Q4
Percent Relative Error
0
50
100
150
200
5
15
30
45
60
75
90
Resampling percentage

Figure S1. Coverage depth saturation curve for all genes with detectable levels of expression in nurses and foragers. RSeQC calculates the relative error for each gene using specified proportions (subsets) of the reads assigned to that gene. These subsets are represented in the x-axis, while the y-axis shows how the estimated distribution of the resampled data deviates from real expression values. The subplots are divided based on the level of expression of their constituent genes (Q1 – lowest 25%, Q4 – highest 25%). Regardless of the level of expression, the percent relative error converges on a stable and accurate representation of the true data, indicating that the read coverage was adequate for differential expression analyses.

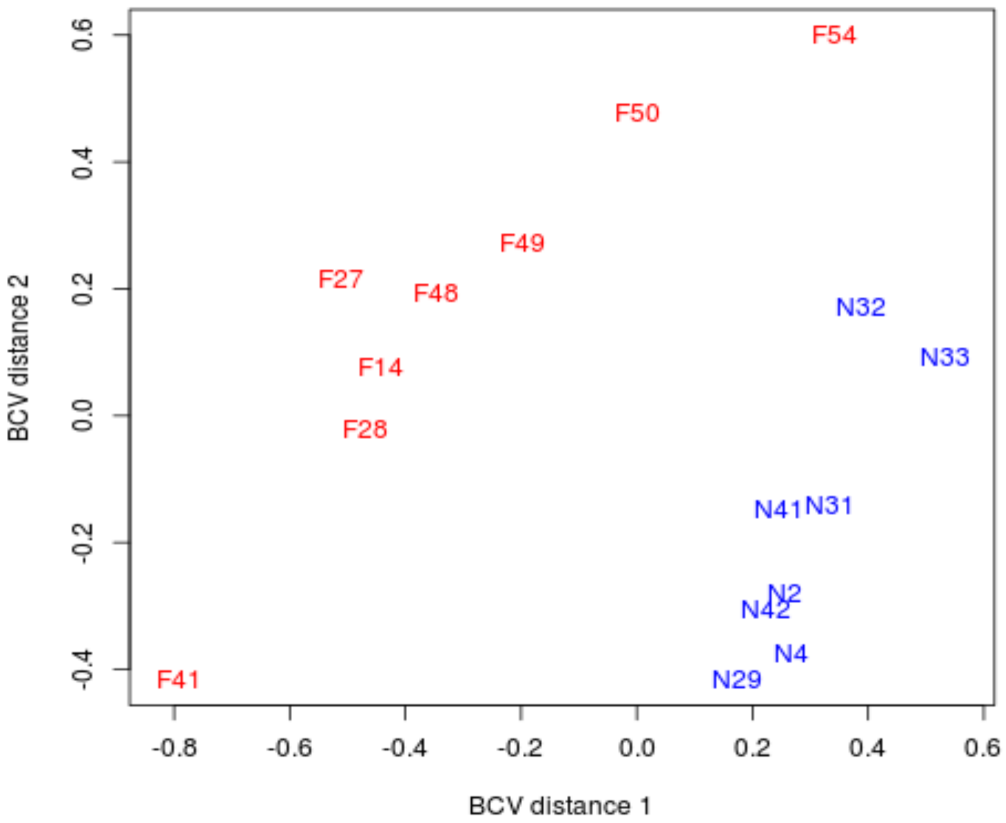

Figure S2. Multidimensional Scaling (MDS) plot of gene expression profiles for all samples shows a higher degree of variation within forager samples than within nurse samples. The distance on the plot between each pair of samples represents the biological coefficient of variation BCV (the square root of the dispersion parameter under the negative binomial distribution for the 500 genes with the largest absolute log-fold-changes of expression between samples).

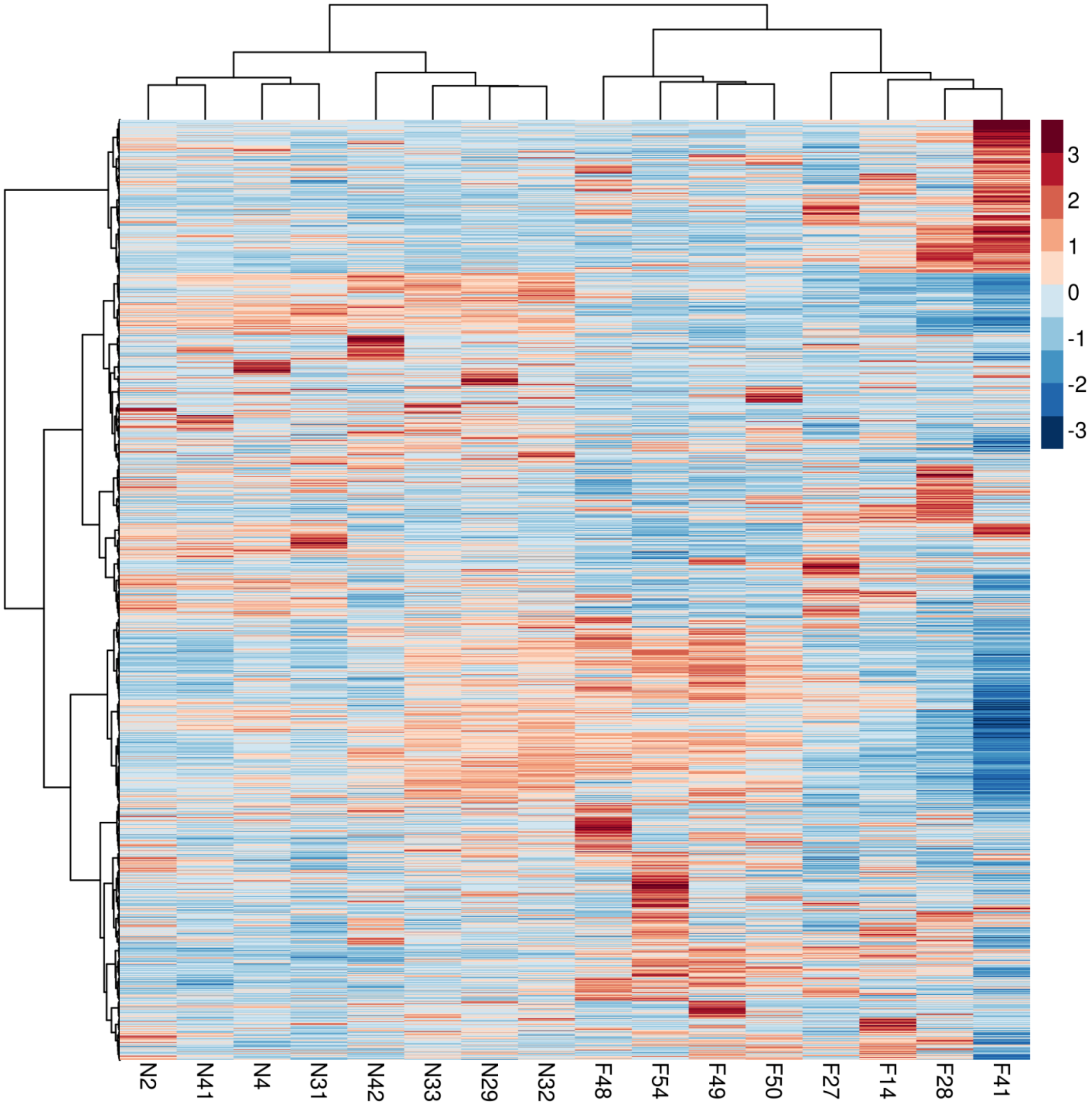

Figure S3. Heatmap for the hierarchical clustering of the gene expression profiles of 16 honey bees. Rows correspond to the 12,453 genes with detectable levels of expression. Columns represent samples. The clustering identified two separate groups of transcription profiles, supporting the clear distinction between the two age-related behavioral groups, nurses and foragers.

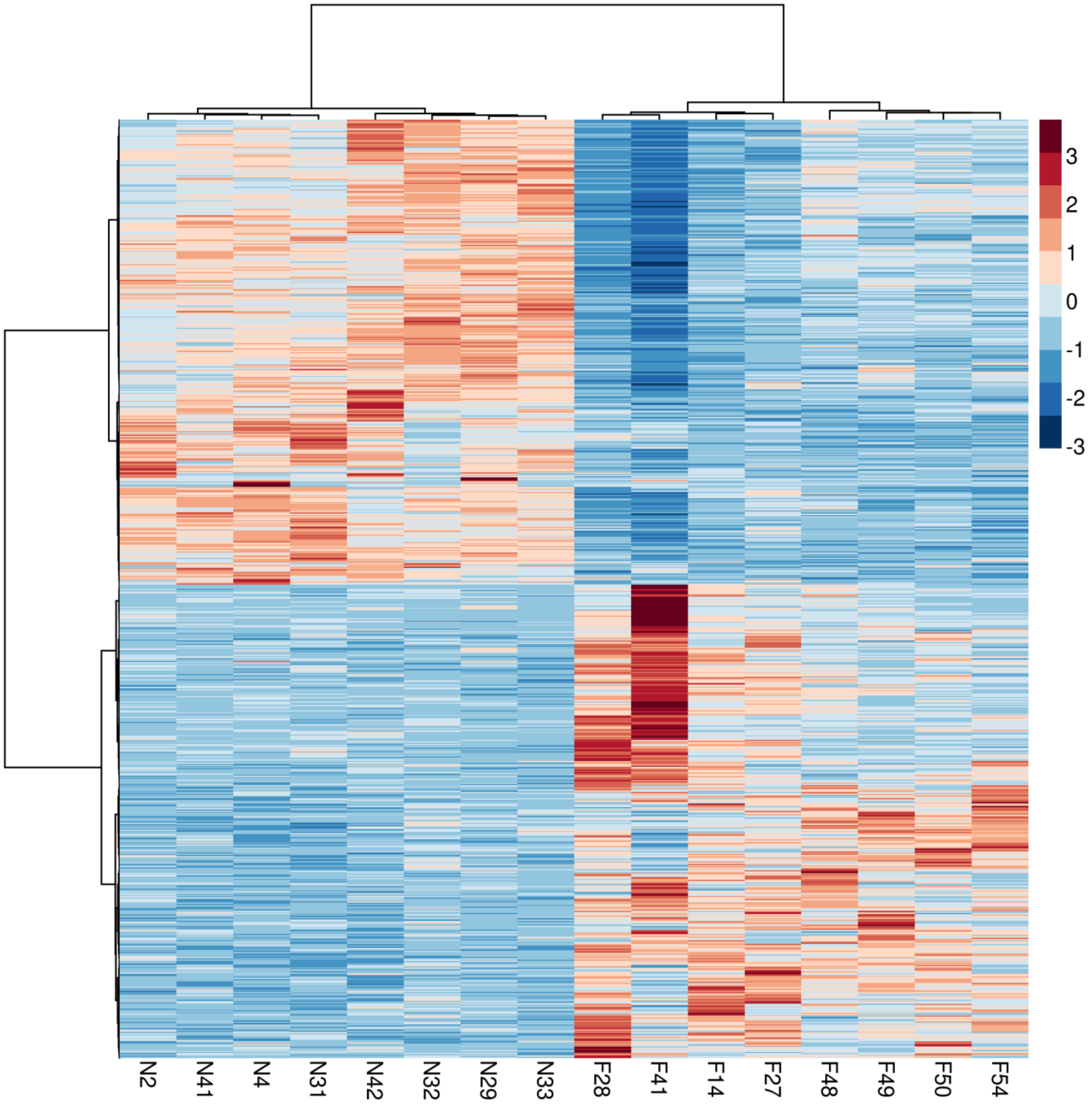

Figure S4. Heatmap for the hierarchical clustering of the gene expression profiles of 16 honey bees. Rows correspond to the 1,058 DEGs and columns represent samples. The clustering shows a clear separation in gene expression pattern between nurses and foragers.

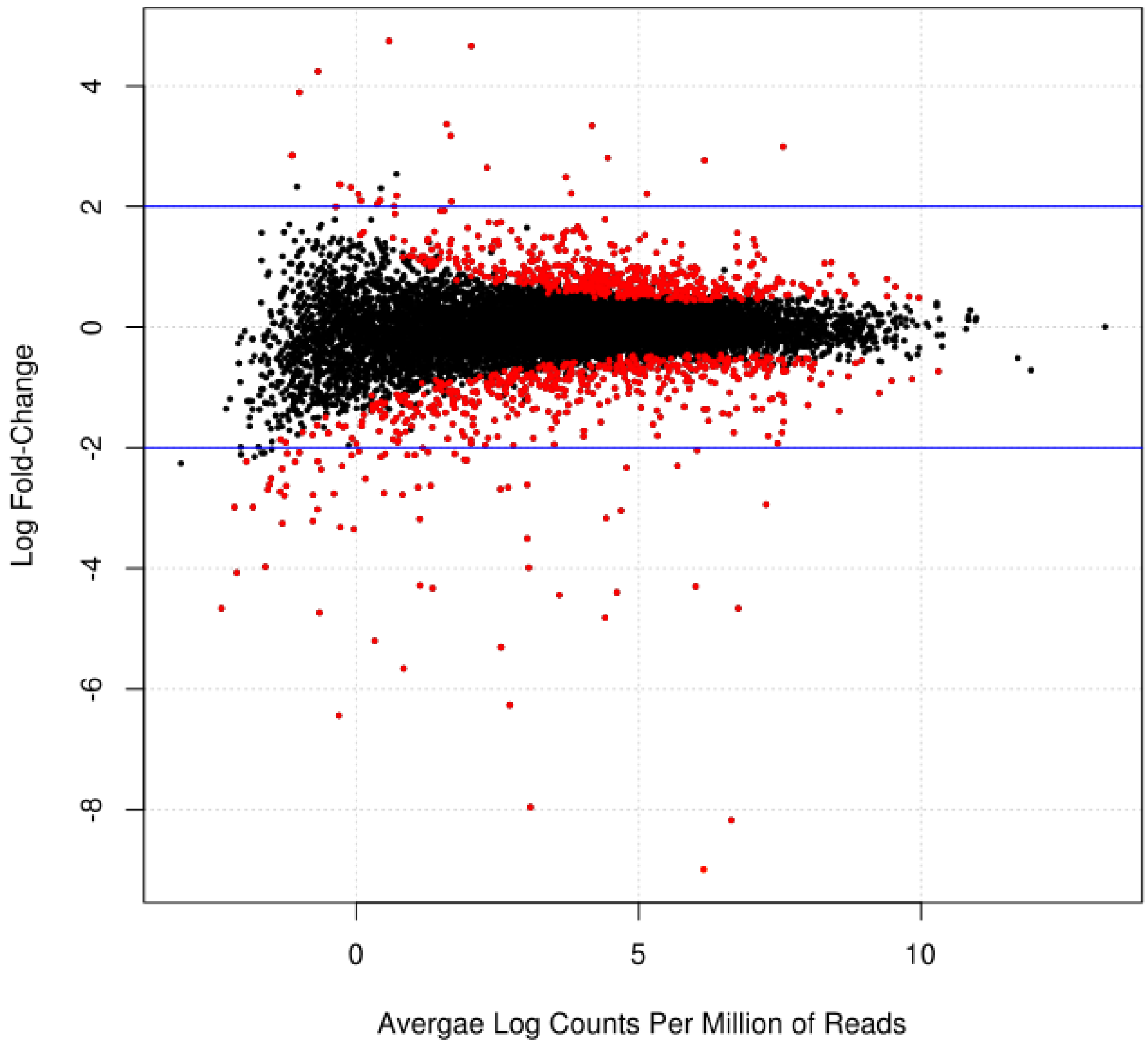

Figure S5. Plot showing the relationship between differential gene expression and read coverage. Differentially expressed genes (FDR<0.05) are in red and non-differentially expressed genes are in black. The two horizontal blue lines show 4-fold changes (log-FC of 2).

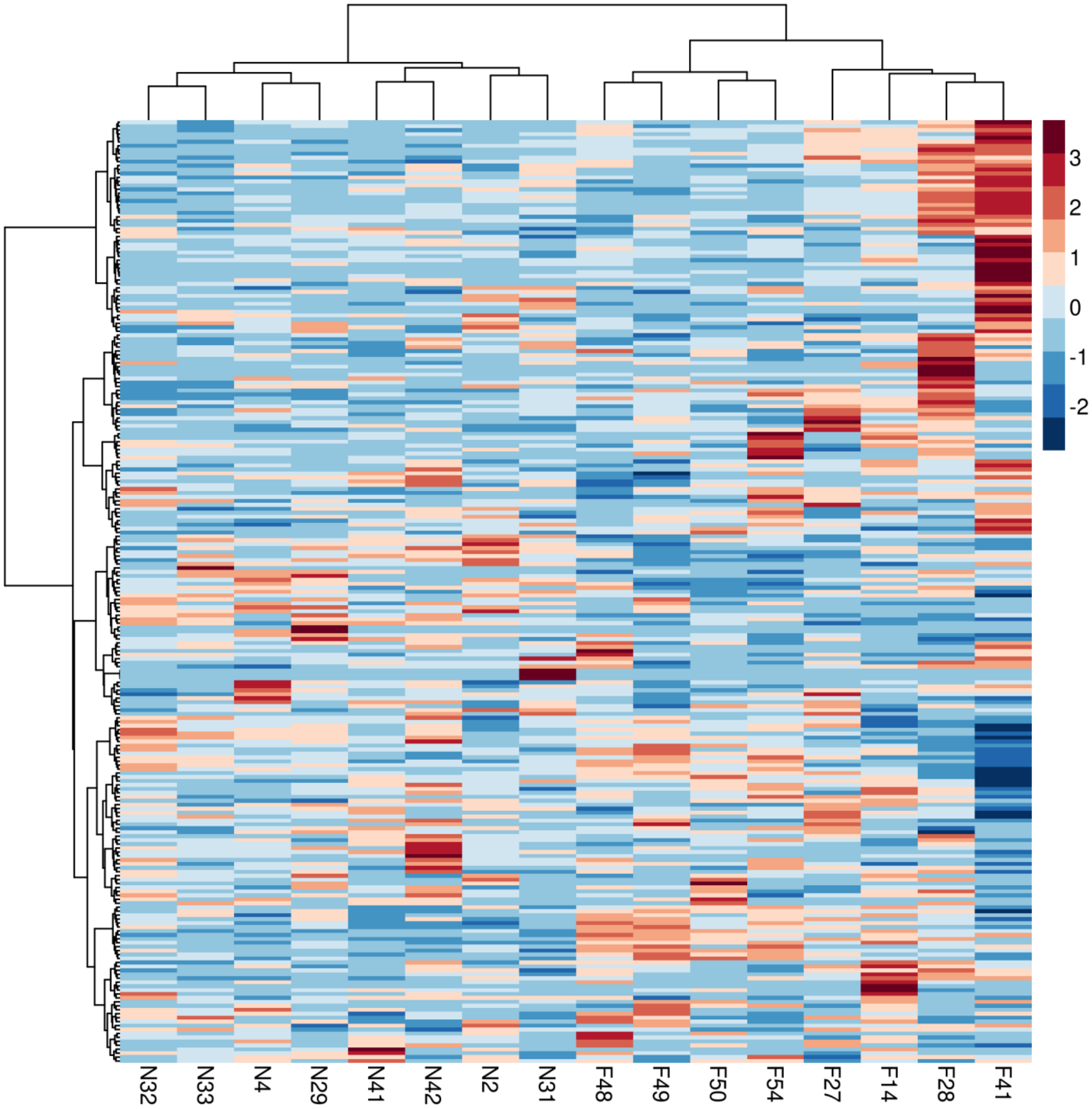

Figure S6. Heatmap for the hierarchical clustering of the expression data of the 239 honey bee TFs with detectable levels of brain expression. Rows represent the 239 TF genesand columns represent nurse (labeled 'N') and forager (labeled 'F') samples.

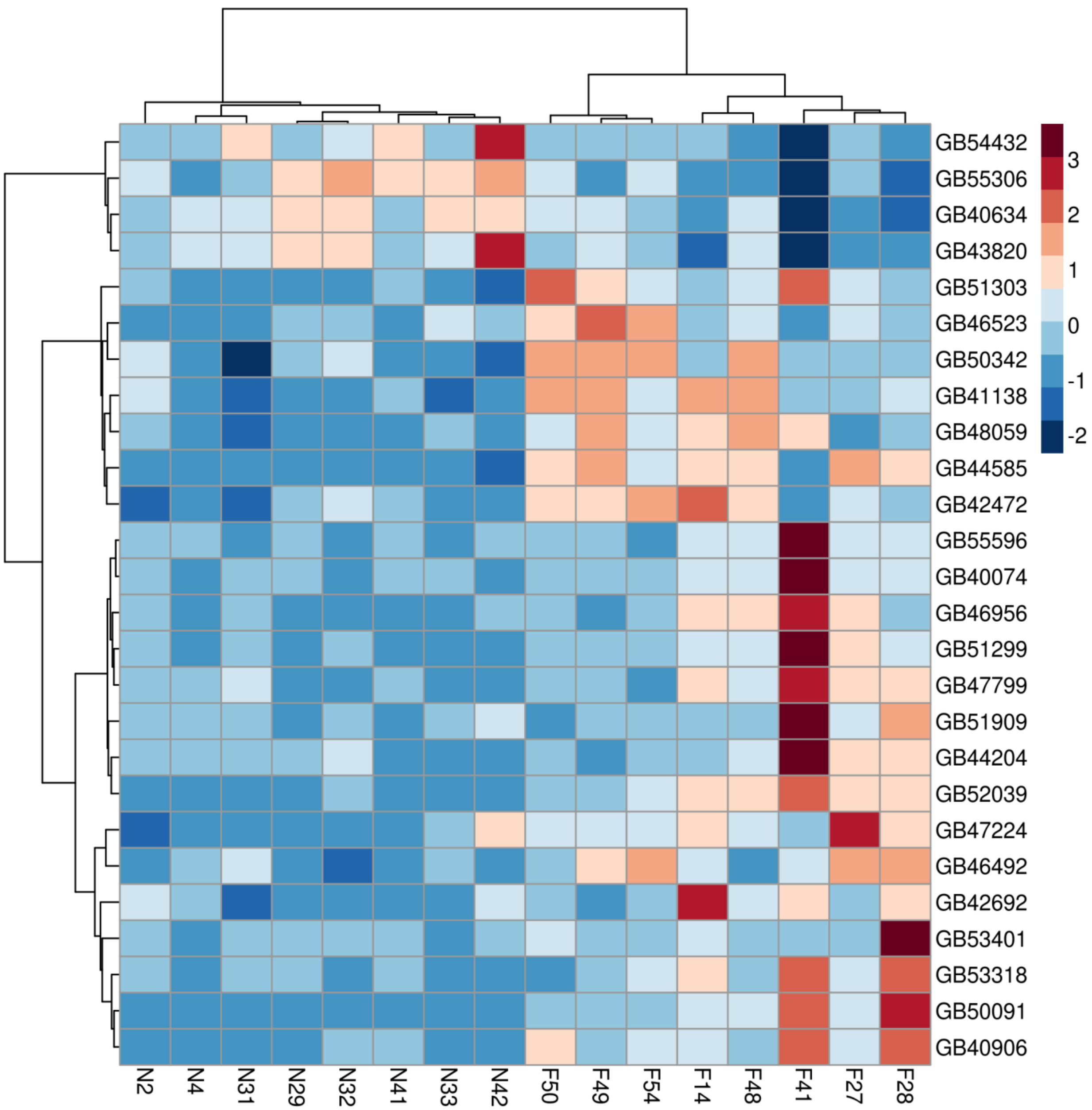

Figure S7. Heatmap for the hierarchical clustering of the expression data of 26 differentially expressed honey bee *Apis mellifera* TF genes. 22 TF genes are upregulated in foragers, and 4 TF genes are upregulated in nurses. Rows represent the 26 TF genes. Columns represent nurse ('N') and forager ('F') samples.

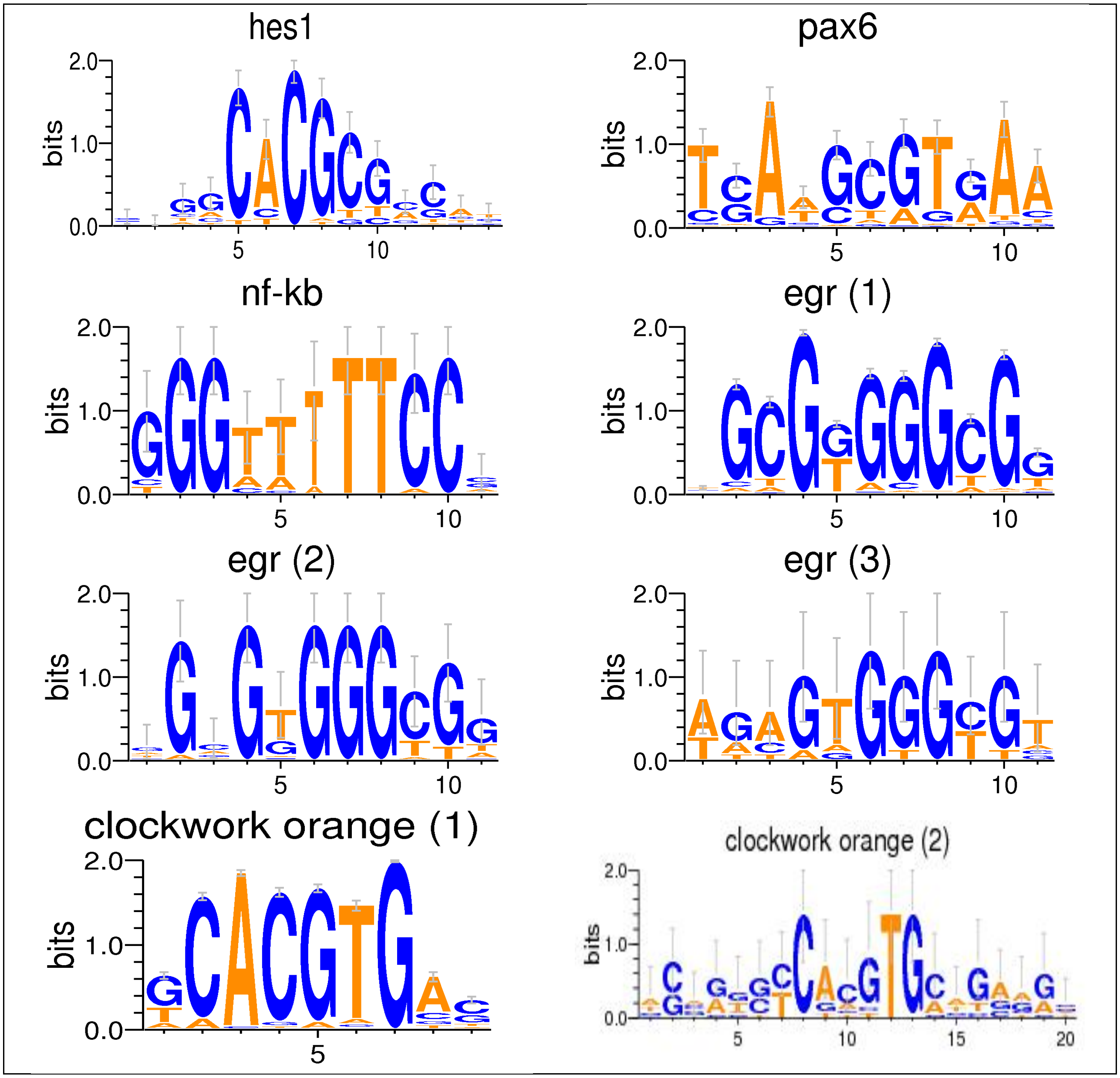

Figure S8. Sequence logos for the position weight matrix (PWM) of the five TFs (*hes1*, *pax6*, *nf-kb*, *egr* and *clockwork orange)* predicted to co-regulate nearly half of all forager upregulated genes. The TFs *egr* and *clockwork orange* have three and two PWMs, respectively. As shown the logos, the motifs of many of these TFs are G/C rich.

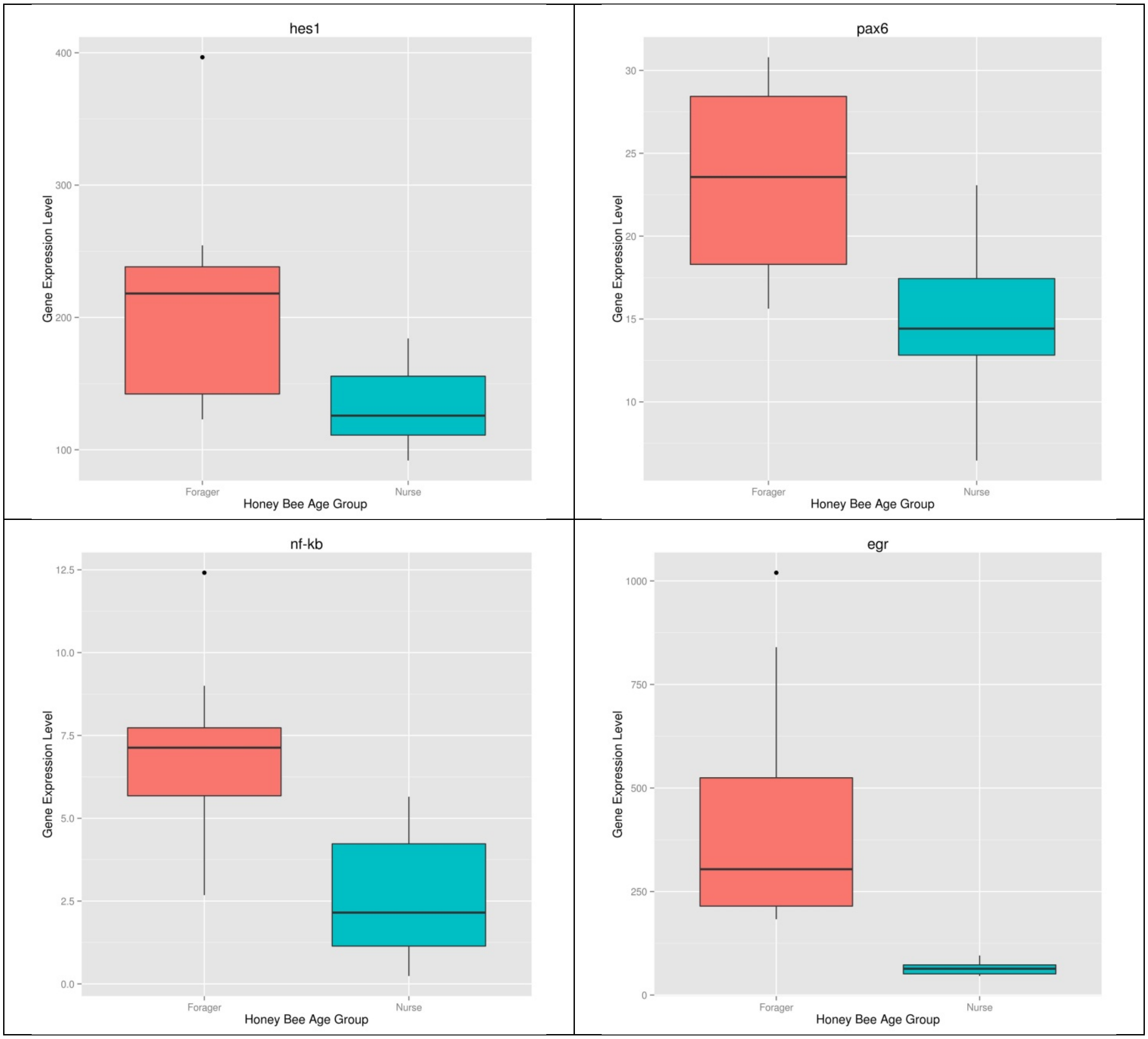

hes1
pax6
nf-kb
egr
Gene Expression Level
Honey Bee Age Group
Forager
Nurse

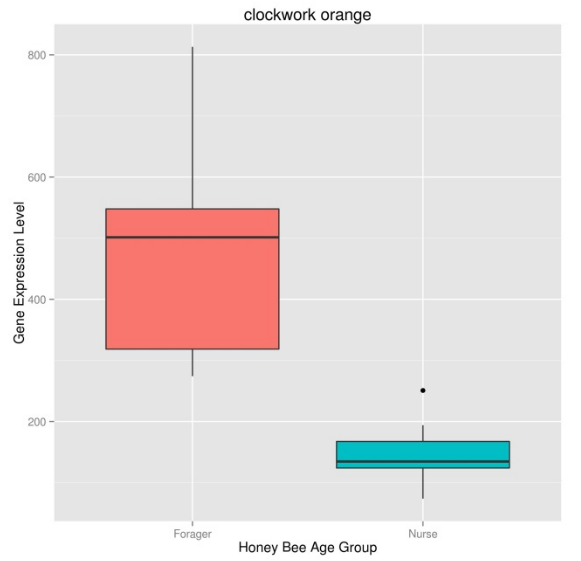

Figure S9. Boxplots for the expression levels of the five TFs (*hes1*, *pax6*, *nf-kb*, *egr* and *clockwork orange)* predicted to co-regulate nearly half of the forager upregulated genes.

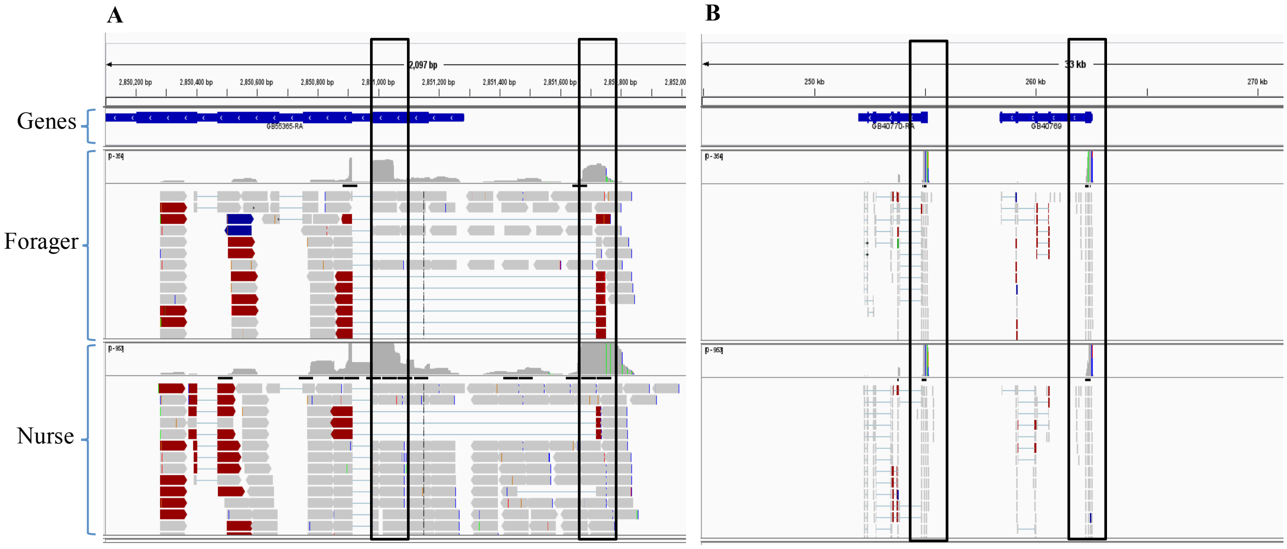

Figure S10. Identification of gene TSSs and related promoters. CAGE paired-end reads were mapped to the reference genome and viewed in reference to the newest honey bee gene annotation (OGS v3.2) to provide accurate identification of gene TSSs and related promoters. We identified different types of transcription start sites (TSS) based on the number of TSSs. Some genes have multiple TSSs (A) (gene ID: GB55365) while other genes use single TSSs (B) (gene IDs: GB40769 and GB40770).

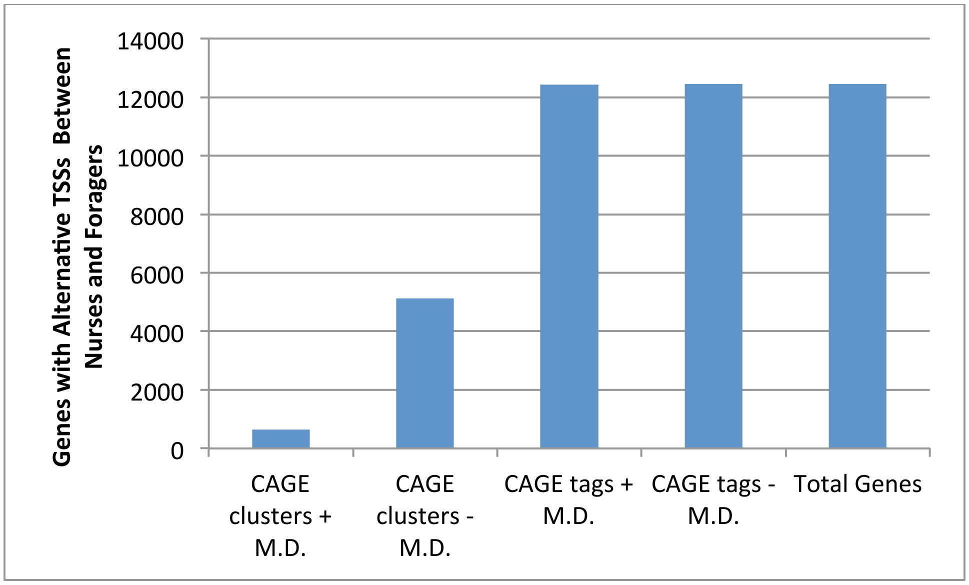

Figure S11. Effect of analysis technique on alternative TSS identification. Displays the influence of analysis technique on the identification of genes with alternative TSSs, using CAGE clusters vs. individual tags and with or without requiring a mutual distance (M.D.) of 100 bp.

Supplementary Tables

Table S1:

A) Relationship between RNA quality and CAGEscan reads.

Sample Name	260/230	260/280	Conc. (ng/µl)	Yield (ng)	RIN value	CAGEscan Reads
**N2**	1.48	2.05	31	**1540**	5.5	14,172,924
**N4**	0.91	2.07	43	**2126**	5.3	12,209,666
**N29**	1.47	2.08	29	**1458**	4.2	10,652,219
**N31**	1.87	1.98	35	**1746**	5.5	10,424,626
**N32**	0.42	1.88	27	**1342**	3.8	11,031,651
**N33**	0.61	2.11	30	**1506**	4.5	10,998,419
**N41**	1.70	1.96	37	**1826**	5.5	12,987,132
**N42**	0.34	2.06	36	**1786**	6.7	10,126,459
**F14**	0.56	1.89	30	**1510**	6.0	5,449,888
**F27**	0.77	1.85	34	**1690**	6.4	3,586,235
**F28**	0.58	1.69	27	**1348**	6.9	7,725,895
**F41**	0.51	2.14	28	**1424**	7.7	9,237,207
**F48**	0.57	2.17	36	**1778**	8.0	2,936,239
**F49**	0.47	2.28	37	**1866**	5.8	4,355,366
**F50**	1.16	2.28	33	**1652**	7.6	4,307,121
**F54**	0.45	2.46	34	**1710**	5.8	2,348,738

This table depicts the Nanodrop quality scores, RNA concentration/total quantity, and Bioanalyzer RIN values of each sample used for CAGEscan. Although there is a high degree of variability in RIN score between individual samples, it is unlikely that this reflects RNA degradation that would adversely affect CAGEscan sequencing for the following reasons:

1) The insect 28s ribosomal subunit can dissociate at the temperatures employed by the Bioanalyzer, which can cause spurious RIN values. An examination of the Bioanalyzer electropherograms revealed that this was, in fact, the case (Supplementary Figure S1B).
2) If RIN score was reflective of the RNA quality in these samples, then a positive correlation should be expected to exist between RIN and sequencing/mapping efficiency. However, no such relationship was found ($p>0.25$, Spearman Rank Correlation). While a modestly significant correlation was found between RIN score and the percentage of

CAGE tags successfully mapped (p=0.016), RIN score was inversely (rather than positively) related to CAGE mapping success (r= -0.59).

B) Sample Bioanalyzer Trace.

Note the bimodal peak identified as the 18s rRNA (rRNA1), and the lack of a peak corresponding to the 28s rRNA. This indicates that the 28s rRNA dissociated and comigrated with the 18s rRNA. Since calculating the RIN value depends on the proper identification of these rRNAs, this disassociation would result in the RIN giving an inaccurate representation of RNA quality.

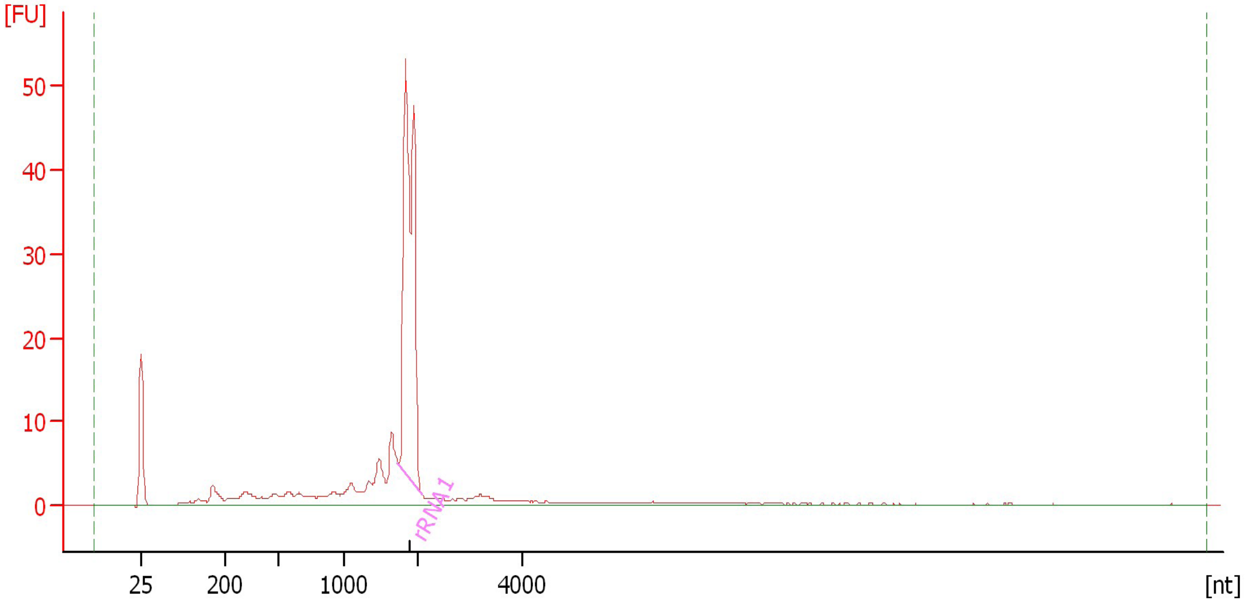

Figure S1B. Bioanalyzer electropherogram for honey bee RNA. The electropherogram does not indicate that any RNA degradation occurred (PMID: 21067419, PMID: 24862828).

Table S2:

Number of reads obtained for each sequenced sample. This table shows general information about the number of reads obtained for each sample in forager and nurse libraries.

Sample	Sample Name	NanoCAGE Library	Total Number of Paired-end Reads
1	F14	Forager	5,449,888
2	F27		3,586,235
3	F28		7,725,895
4	F41		9,237,207
5	F48		2,936,239
6	F49		4,355,366
7	F50		4,307,121
8	F54		2,348,738
			**Total = 39,946,689**
1	N2	Nurse	14,172,924
2	N4		12,209,666
3	N29		10,652,219
4	N31		10,424,626
5	N32		11,031,651
6	N33		10,998,419
7	N41		12,987,132
8	N42		10,126,459
			**Total = 92,603,096**

Table S3:

Mapping statistics of library reads using Tophat. Column 3 shows the number of mapped reads obtained using Tophat when default values for the (average inner distance between mate reads of a pair of reads=20) and (standard deviation of inner distance=50) were used. Column 4 shows the number of mapped reads using Tophat when the estimated values for (average inner distance between mate reads of a pair of reads=588) and (standard deviation of inner distance=767) were used. These values were estimated as follows. We used bowtie2 to map the reads initially. Then, we estimated these values from the distances between mates of the properly mapped paired-end reads. We used these values eventually to map the reads using Tophat. The mapping ratio is shown in column 6.
Note: The values in column 3 may be greater than the original number of reads in column 2. This is because reads are not necessarily paired during the mapping process. Consequently, the number of mapped reads is calculated based on individual mates of paired-reads.

Sample	Total Number of Reads	Total Number of Mapped Reads (using default settings)	Total Number of Mapped Reads (using estimated distances)	Unique Mapped Reads	Mapping Ratio
F14	5,449,888	6,030,292	6,134,426	3,143,064	57.67%
F27	3,586,235	4,002,757	4,071,609	2,157,798	60.16%
F28	7,725,895	7,927,736	8,057,070	4,244,582	54.93%
F41	9,237,207	5,895,966	5,981,393	3,130,408	33.88%
F48	2,936,239	3,361,068	3,416,792	1,798,240	61.24%
F49	4,355,366	5,716,008	5,808,440	3,040,200	69.80%
F50	4,307,121	5,745,833	5,839,990	2,955,469	68.61%
F54	2,348,738	3,021,819	3,070,740	1,623,527	69.12%
N2	14,172,924	15,706,801	15,967,039	8,084,168	57.03%
N4	12,209,666	13,228,888	13,450,770	7,068,767	57.89%
N29	10,652,219	13,739,874	13,981,602	6,949,056	65.23%
N31	10,424,626	12,889,625	13,127,848	6,820,778	65,42%
N32	11,031,651	13,393,432	13,621,784	7,173,714	65.02%
N33	10,998,419	14,417,662	14,663,617	7,503,984	68.22%
N41	12,987,132	15,571,634	15,821,593	8,050,612	61.98%
N42	10,126,459	12,105,027	12,305,292	6,529,766	64.48%

Table S4:

Filtration results on the mapped reads based on quality threshold, mapping both mates of paired-end reads and insertion size. We applied some filtration methods to maintain mapped reads having good mapping quality and were properly paired and within considerable distance between the mates of the pair. First three columns are identical to columns 1, 2 and 4, respectively, of Supplementary Table S3.

Columns Description:

Column1 (Sample): Sample Name
Column2 (Total Number of Paired-End Reads): The original total number of paired end reads we have
Column3 (Total Number of Mapped Reads): The total number of reads mapped by Tophat. The number of mapped reads is calculated based on individual mates of paired-reads because reads are not necessarily paired during the mapping process. For this reason, the numbers in this column may be greater than the original number of reads in column 2.
Column4 (Mapped Reads with Good Mapping Quality): The total number of mapped reads which passed the quality threshold (20)
Column5 (Correctly Mapped Reads): The total number mapped reads which passed the quality threshold (20) and were properly paired during mapping (an example of the incorrect mapped read is when mates are mapped in the wrong direction)
Column6 (Within Insertion Size 1513): The total number mapped reads which passed the quality threshold (20) and were properly paired during mapping and have insertion size <1513
Column 7 (Unique Mapped Reads): The unique number of mapped reads in column 6

Sample	Total Number of Paired-End Reads	Total Number of Mapped Reads	Mapped Reads with Good Mapping Quality	Correctly Mapped Reads	Within Insertion Size (1513)*	Unique Mapped Reads
F14	5,449,888	6,134,426	5,235,317	4,132,273	3,321,089	1,661,003
F27	3,586,235	4,071,609	3,643,431	2,886,570	2,321,172	1,160,788
F28	7,725,895	8,057,070	7,043,813	5,571,558	4,507,082	2,254,095
F41	9,237,207	5,981,393	5,008,184	3,757,953	3,204,608	1,602,772
F48	2,936,239	3,416,792	3,029,380	2,367,407	1,914,907	957,521
F49	4,355,366	5,808,440	5,155,830	4,120,182	3,290,889	1,645,762
F50	4,307,121	5,839,990	5,001,987	3,976,839	3,151,237	1,575,704
F54	2,348,738	3,070,740	2,741,950	2,152,211	1,717,604	858,840
N2	14,172,924	15,967,039	13,470,127	10,603,418	8,192,48q7	4,096,705
N4	12,209,666	13,450,770	11,815,466	9,345,058	7,329,738	3,665,374
N29	10,652,219	13,981,602	11,670,196	9,301,672	7,308,576	3,654,964

N31	10,424,626	13,127,848	11,581,843	9,301,324	7,174,563	3,587,528
N32	11,031,651	13,621,784	11,967,574	9,366,077	7,295,335	3,648,373
N33	10,998,419	14,663,617	12,600,241	9,984,766	7,710,155	3,855,377
N41	12,987,132	15,821,593	13,401,785	10,486,051	8,014,535	4,007,666
N42	10,126,459	12,305,292	10,788,376	8,302,999	6,482,828	3,241,663

*1513 is the template length which is:
(Average insertion size+ standard deviation + paired read lengths)
i.e. (588 + 767+ 79*2) = 1513

Table S5:

Statistics of the association between CAGE tags and OGS v3.2 genes.

Sample	Total CAGE Tags in *(Positive)* Strand	CAGE Tags in *(Positive)* Strand which could be Associated to Genes	Percentage of CAGE Tags Associated to Genes to the Total CAGE Tags in *(Positive)* Strand	Total CAGE Tags in *(Negative)* Strand	CAGE Tags in *(Negative)* Strand which could be Associated to Genes	Percentage of CAGE Tags Associated to Genes to the Total CAGE Tags in *(Negative)* Strand	Percentage of CAGE Tags Associated to Genes to the Total CAGE Tags in *(Both Strands)*
**Forager**							
F14	744,521	636,437	85.48%	616,553	552,489	89.60%	87.35%
F27	516,490	448,612	86.85%	429,271	394,422	91.88%	89.14%
F28	998,448	812,140	81.34%	795,356	718,421	90.32%	85.32%
F41	704,367	557,640	79.16%	534,496	466,164	87.21%	82.64%
F48	425,089	372,402	87.60%	369,030	334,903	90.75%	89.07%
F49	729,469	641,975	88.00%	634,005	578,035	91.17%	89.48%
F50	695,749	601,638	86.47%	609,247	553,558	90.85%	88.52%
F54	381,211	330,342	86.65%	336,965	305,528	90.67%	88.54%
**Nurse**							
N2	1,801,355	1,553,886	86.26%	1,540,413	1,394,063	90.49%	88.22%
N4	1,602,326	1,385,226	86.45%	1,345,999	1,234,192	91.69%	88.84%
N29	1,586,950	1,382,406	87.11%	1,362,343	1,249,877	91.74%	89.25%
N31	1,549,518	1,323,682	85.42%	1,280,533	1,190,560	92.97%	88.84%
N32	1,597,414	1,399,339	87.60%	1,397,086	1,283,929	91.90%	89.61%
N33	1,677,454	1,468,739	87.55%	1,464,781	1,343,610	91.72%	89.50%
N41	1,746,244	1,503,673	86.10%	1,478,731	1,350,915	91.35%	88.52%
N42	1,384,485	1,176,963	85.01%	1,200,185	1,100,572	91.70%	88.12%

Table S6:

Comparison of Major Findings with/Without the Outlier F41

	Forager Upregulated Genes	Nurse Upregulated Genes	Forager Upregulated TFs	Nurse Upregulated TFs	Key Regulators
With Sample F41	534	524	22	4	5
Without Sample F41	520	660	18	3	5
Percent Concordance	77.7%	95.2%	82%	75%	100%

Removing the outlier F41 had little impact on subsequent analyses.

Table S7:

Assessing Potential Hypopharyngeal Gland Contamination of Gene Expression

Nurse HPG vs Nurse Brain				Forager HPG vs Forager Brain			
Top	Log_2 Fold Change	HPG Upregulated Genes	CAGEscan DEG Overlap	Top3	Log_2 Fold Change	HPG Upregulated Genes	CAGEscan DEG Overlap
1%	>6.5	181	0	1%	>7.25	106	5
5%	>3.0	591	17	5%	>3.0	534	19
10%	>2.0	1067	34	10%	>2.0	1066	55
20%	>1.0	2133	74	20%	>1.0	2132	105

Comparison of nurse and forager HPG upregulated genes with their respective CAGEscan counterparts.

Table S8:

Adapters and barcodes used in nanoCAGE libraries.

Adapters ligated to the 5’ end of the CAGE tags:

Strand	Sequence (5'-3')
5’ end	<Barcode>NNNNNNNNTATA(rG)(rG)(rG)

5’ end barcodes used for nanoCAGE libraries:

Sample Name	NanoCAGE Library	Barcode
F14	Forager	ACAGAT
F27		ATCGTG
F28		CACGAT
F41		CACTGA
F48		CTGACG
F49		GAGTGA
F50		GTATAC
F54		TCGAGC
N2	Nurse	ACAGAT
N4		ATCGTG
N29		CACGAT
N31		CACTGA
N32		CTGACG
N33		GAGTGA
N41		GTATAC
N42		TCGAGC

Supplementary Datasets

Supplementary Dataset 1. List of 1,058 differentially expressed genes (DEGs) between nurses and foragers. 534 DEGs were upregulated in foragers and 524 DEGs were upregulated in nurses.

Supplementary Dataset 2. Similarity of DEGs between CAGEscan and previous studies.

Supplementary Dataset 3. Gene Ontology (GO) analysis based on the category-frequency of enriched GOs (using CateGOrizer).

Supplementary Dataset 4. Gene Ontology (GO) analysis based on the frequency of GO terms associated with forager/nurse DEG list relative to genomic background using Fisher's exact test, followed by FDR correction for multiple testing (FDR < 0.05).

Supplementary Dataset 5. List of 26 Transcription Factors were differentially expressed between nurses and foragers, with 4 and 22 upregulated TFs in each context, respectively. These TFs were identified as Key Regulators of Behavioral Maturation.

Supplementary Dataset 6. G/C content analysis for the promoters of DEGs.

Supplementary Dataset 7. Number of Forager DEG Genes whose promoters were enriched for motifs of TFs.

Supplementary Dataset 8. Distance Between nurse and forager TSS (bps) for all 1,058 DEGs.

Supplementary Dataset 9. Gene Ontology (GO) analysis of the 646 genes with alternative TSSs using (DAVID GO analysis tool).
